# Formulation and Ripening Duration of Italian-Style Ostrich Salami: Impact on Physicochemical Quality and Sensory Traits

**DOI:** 10.3390/foods15142462

**Published:** 2026-07-11

**Authors:** Enrico Novelli, Marco Cullere, Louwrens Hoffman, Stefania Balzan, Antonella Dalle Zotte

**Affiliations:** 1 Department of Comparative Biomedicine and Food Science, University of Padova, Agripolis, Viale dell’Università, 16, 35020 Legnaro, PD, Italy; enrico.novelli@unipd.it (E.N.); stefania.balzan@unipd.it (S.B.); 2 Department of Animal Medicine, Production and Health, University of Padova, Agripolis, Viale dell’Università, 16, 35020 Legnaro, PD, Italy; marco.cullere@unipd.it; 3 Centre for Nutrition and Food Sciences, Queensland Alliance for Agriculture and Food Innovation, The University of Queensland, St. Lucia, Brisbane, QLD 4072, Australia; louwrens.hoffman@uq.edu.au; 4 Department of Animal Sciences, Faculty of AgriSciences, University of Stellenbosch, Stellenbosch 7600, South Africa

**Keywords:** *Struthio camelus*, fat inclusion level, salt inclusion level, microbial starters, dry-fermented sausage, long ripening, free fatty acids, non-protein nitrogen, primary and secondary lipid oxidation products

## Abstract

The present research investigated the effects of two pork back-fat concentrations (30% fat, FAT30, and 40% fat, FAT40), two sodium chloride levels (2.4% and 2.6%), and two starter culture combinations (*Lactobacillus curvatus*/*Staphylococcus xylosus*; LAB6, and *Lactobacillus sakei*/*Staphylococcus xylosus*; LAB8) on ripened ostrich salami. Salami samples were formulated without nitrite and nitrate, which aligns with consumer demands for healthier, cleaner-label meat products. It is specified that the present experiment is structured with a single-batch-per-treatment combination: this was due to structural processing limitations in the production facility, which was an artisanal laboratory and not an industry plant. After 10 weeks of ripening, FAT30 salami showed higher values of pH, salt content, water-phase salt (WPS), α-tocopherol, free fatty acids (FFA), and secondary lipid oxidation products (TBARS) compared with FAT40 salami. Conversely, FAT40 salami exhibited higher water activity (a_w_), moisture-to-protein ratio (M:P), conjugated dienes (CD; primary lipid oxidation products), and non-protein nitrogen (NPN) than FAT30 salami. Both NaCl concentration and starter culture type influenced several of the measured variables. Specifically, salami containing 2.4% salt exhibited higher FFA and CD values than the formulation containing 2.6% salt. Likewise, the LAB8 starter culture resulted in higher CD and NPN levels compared with LAB6. Fat inclusion level significantly affected sensory characteristics. FAT40 salami exhibited greater intensities of gamy, metallic, fatty, and moldy flavors, as well as higher overall off-flavor intensity, tenderness, and juiciness. In contrast, FAT30 salami was characterized by greater cohesiveness and a more pronounced ripening flavor. The 2.6% sodium chloride treatment resulted in greater color homogeneity, higher odor intensity, and stronger rancid notes, while reducing the perception of metallic, fatty, and moldy flavors compared with the 2.4% treatment. Salami inoculated with LAB6 exhibited a higher intensity of off-flavors than the formulation produced with LAB8. Moreover, several significant interactions among the three experimental factors were observed. After 20 weeks of ripening, the effects observed after 10 weeks for most physicochemical parameters were largely maintained. However, FFA and CD concentrations (both below the limit of quantification) no longer differed between the two fat inclusion levels. Sensory evaluation revealed that the differences between FAT30 and FAT40 in undesirable flavor attributes disappeared over time, whereas the perception of ripening and maturity became even more pronounced in FAT30 salami. Regarding FA composition, FAT30 salami contained higher proportions of saturated FA and polyunsaturated FA, whereas FAT40 salami was characterized by a higher monounsaturated FA content and more favorable lipid quality indices.

## 1. Introduction

Mediterranean countries have been producing natural fermented sausages since Roman times, and production gradually spread across Europe [1]. Regional ingredients, climates, and techniques yield traditional fermented foods with unique sensory profiles. In Italy, localized salami types arise from complex biochemical and physical reactions driven by the spontaneous growth of indigenous batter microflora during ripening [2]. By Italian law, salami is a cured meat product made from minced striated muscle (chiefly pork) and variable proportions of pork fat, seasoned with salt (NaCl), iodized salt, potassium chloride (KCl), or sodium substitutes. Stuffed into natural or artificial casings, it is dried and ripened to promote natural fermentation and enzymatic processes, ensuring room-temperature shelf stability and its characteristic sensory profile. On the market, salami must have a pH ≤ 4.9 [3]. Among other meats, ostrich meat represents a viable alternative ingredient, being a gourmet, exotic, and healthy red meat with water, protein, amino acids, and mineral contents comparable to those of beef and chicken [4], low intramuscular fat content (around 2% m. *iliofibularis*), and a healthy fatty acid (FA) profile that can be further enhanced by dietary interventions [5,6]. In 2024, Italian salami production reached 130,400 tons, with 40% destined for export, primarily to France, Germany, the United States and the United Kingdom [7]. A survey of 154 small and medium-sized companies across five provinces in northern Italy revealed that all (except one) used pork exclusively, with lean meat in the batter ranging from 60% to 91% depending on the salami typology [8]. The production process involves ingredient formulation, fermentation, and subsequent ripening. Three critical formulation ingredients are recognized: added fat, salt and nitrate/nitrite salts. Fat content (10–40%) must be firm, with a high melting point and low PUFA content to prevent rancidity and oily exudation [9,10]. Beyond sensory contributions, minced fat facilitates continuous moisture release during drying and ripening. Consequently, reducing fat directly affects the sensory quality and consumer acceptability of salami [11,12]. NaCl serves three key functions: (i) microbiological stabilization through water activity reduction and dehydration; (ii) enhancement of water-binding capacity through myofibrillar protein solubilization and improving meat-fat binding in restructured meat; and (iii) flavor and palatability enhancement by attenuating bitterness and sweetness [13,14,15]. A concentration of 2.5% NaCl represents the lower limit for good-quality salami, while >2% is required for correct fermentation regulation [16]. Nitrite (NO_2_^−^) and nitrate (NO_3_^−^) are widely used as antimicrobials in cured meats to inhibit *Clostridium botulinum*, limit growth of spoiling organisms, retard lipid peroxidation, and develop characteristic flavor and color [17,18,19] at the maximum amounts of 80 and 90 mg/kg, respectively (values referred at the level of manufacturing and expressed as NO_2_ and NO_3_ ion) [20]. However, these compounds are precursors of carcinogenic N-nitroso compounds, raising concerns about their role in cancer [21,22]. Meanwhile, Italian and European consumer demand for traditional fermented products, such as artisanal salami, continues to grow [23] despite their high fat and NaCl content, which are associated with obesity and cardiovascular diseases [24]. Reducing fat and salt in dry-fermented salami, however, may alter water activity and accelerate weight loss [25]. In European countries, two production methods can be clearly distinguished: dry curing in southern Europe (typical of Mediterranean countries) and wet/pickled curing in northern Europe. In every country, there are examples of naturally fermented sausages that, as is also the case in Italy, currently represent only a small share of the dry-cured sausage market. However, they are no less important in terms of production tradition and sensory characteristics, and they continue to be of great interest to consumers. As a general description, the Italian traditional salami is made using meat of Italian heavy pigs, whose carcasses must weigh between 110 kg and 180 kg; the typical taste comes from long ripening, a reduced acidification (not acid product), not salty taste, accentuated and uniform red color and superficial microbial growth with molds able to impart agreeable and traditional aspects and flavor [26]. The batter is made by mixing pork, lean and fat in variable proportions, with equally variable grinding grain, but generally above 5–6 mm with the addition of salt, black pepper, white or red wine depending on tradition, sometimes crushed garlic, and often with the addition of nitrate/nitrite salts and stuffed using natural casing. To avoid intense acid taste, the fermentation step is generally conducted at temperature lower than 20 °C and with relative humidity that is managed in order to allow important weight loss and prevent the formation of external crust. In the ripening step, the temperature is further decreased with the aim of achieving a long ripening period that, according to the salami size, can be also of several months, obtaining a total weight loss of 30% or more depending on the percentage of fat employed in the formulation. Historically driven by indigenous microbiota, this spontaneous fermentation is selectively promoted by NaCl, nitrate/nitrite salts, and anaerobic conditions, which favor the growth of beneficial lactic acid bacteria (LAB) and coagulase-negative cocci (CNC), of which *Staphylococcus xylosus* and *Staphylococcus carnosus* are the most common species identified from traditional products [27]. The use of commercial starter cultures has grown to standardize quality, reduce ripening time and, most importantly, for safety reasons, though often at the expense of sensory uniqueness compared to artisan dry-fermented salami [28]. Consequently, research has focused on identifying autochthonous starter cultures better suited to specific products [29,30,31]. Starter cultures, typically combining LAB and CNC, modify sensory properties through metabolic activity. LAB ferment carbohydrates, lowering pH to inhibit pathogens and extend shelf-life, while also promoting muscle protein coagulation and improving slice stability, firmness, and cohesiveness. CNC contribute to flavor and aroma formation, color stabilization via nitrate/nitrite reduction, peroxide decomposition, proteolysis and lipolysis [32]. European Mediterranean consumers traditionally prefer low-acid fermented sausages (pH 5.3 to 6.2), characterized by medium-long processing times and significant regional diversity [33,34]. In southern Europe, *Lactobacillus* (*Lb*.) *sakei*, *Lb. curvatus* and *Lb. plantarum* are the predominant LAB species, being more competitive than other lactobacilli, whereas *Staphylococcus xylosus* is one of the most prevalent CNC [29]. *Lb. sakei* and *Lb. curvatus*, isolated from meat, possess proteolytic activity on muscle proteins, playing a key role in amino acid generation [1,35].

Studies have investigated the use of ostrich meat in fermented sausage production. Italian-style salami produced with ostrich meat using three starter culture combinations: *Lb. sakei* DF 109 + *Micrococcus* sp. MC 50 (batch 1), *Lb. curvatus* DF 38 + *Micrococcus* sp. MC 50 (batch 2), and a combination of all three strains (batch 3), as well as a glucono-delta-lactone (GdL) acidified control (1% *w*/*w*) have been reported [36]. Final pH ranged from 4.87 to 4.92 (5.23 for GdL), with batch 2 receiving the best sensory scores, and GdL the worst. Ostrich sausages fermented without starter cultures showed higher final pH and greater *Pseudomonas* and *Micrococcaceae* counts compared to deer and pork sausages, likely due to the naturally high pH of fresh ostrich meat [37]. Compared to pure pork fermented sausage, no differences were noted in the aroma, flavor, or texture of three batches containing 19.1%, 38.3% and 57.6% ostrich meat [38]. However, the highest ostrich meat content produced a significantly darker color, attributed to the high myoglobin content in ostrich meat.

Based on the above-mentioned considerations for the nutritional and technological characters of ostrich meat, this study primarily aimed at evaluating the impact of different formulation parameters (two fat levels, two NaCl concentrations, and two starter culture mixtures) on the physicochemical properties, FA composition, and sensory profile of artisanal Italian-style ostrich salami manufactured without nitrate and nitrite salts. Furthermore, the evolution of salami nutritional and sensory profiles through the ripening period was also assessed (at 10 and 20 weeks of ripening). The results of the present manuscript complete the research outcomes of the whole project: in fact, the first part of the study, considering weight loss, proximate composition and cholesterol content, was published by Cullere et al. [39]. The present work was designed as a controlled formulation study rather than a production variability study. Therefore, the main objective was to compare the technological response of different formulations under identical manufacturing conditions while minimizing process variability.

## 2. Materials and Methods

### 2.1. Experimental Design and Dry-Fermented Ostrich Salami Manufacturing

Experimental design and salami preparation were described in detail in the first part of the study by Cullere et al. [39]. Briefly, a 2 × 2 × 2 design with a total of 8 batches with 9 ostrich salami portions each (approximately 650 g each, fresh weight) using 2 different levels of fresh pork back fat (30% and 40%), NaCl (2.4% and 2.6%) and two different LAB starter cultures (LAB6: *Lactobacillus curvatus*/*Staphylococcus xylosus* + dextrose or LAB8: *Lactobacillus sakei*/*Staphylococcus xylosus* + dextrose) were produced (BIOAGRO S.r.l., Thiene, Italy). Since nitrite/nitrate salts were not used, the employment of starter culture was aimed at obtaining a regular fermentation for the control of spoilage and pathogenic bacteria. The ostrich meat used to produce the salami was obtained from one male ostrich and had the following chemical composition: 24.9 g/100 g dry matter, 21.1 g/100 g protein, 1.5 g/100 g ash, and 3.09 g/100 g lipids (saturated fatty acids: 32.8%, monounsaturated fatty acids: 31.5%, polyunsaturated fatty acids: 21.9%). Using the meat of one ostrich was a specific choice: despite representing a limitation for the external validation and generalization of the results, it helped in controlling raw-material variability within the experiment. This was fundamental in order to adequately understand and interpret results associated with the different experimental factors under investigation. Salami preparation was carried out as follows: ground ostrich meat and fat were divided into two batches according to two different fat inclusion percentages and then carefully mixed. Afterwards, each batch was divided into two equal units and mixed either with 2.4 or 2.6% of NaCl. Then, the following ingredients were added to each batch: 0.78% black pepper, 0.009% cinnamon, 0.009% cloves, 0.009% nutmeg and 3.55% red wine. Finally, each unit was divided into two equal parts, and each inoculated according to the selected LAB starter culture (1.6 g/kg). Subsequently, the batches were individually mixed again and put in a refrigerated chamber at +4 °C for 12 h.

Afterwards, all batters were stuffed into natural casings (diameter range, 6–8 cm), labeled and fermented for 5 days (relative humidity 65–85% and temperature starting from 19 °C and decreasing by 1 °C/day to 14 °C). Then, the ripening phase was conducted using relative humidity between 70 and 80% and temperature decreased to 12 °C. Ripening of the first group of salami samples (*n* = 4 sausages/treatment) was stopped when the first sample lost up to 35% of its initial weight (at 10 weeks), while the second group of samples (*n* = 5 samples/treatment) was ripened for a further 10 weeks [39]. During drying and ripening, surface mold growth was not prevented, consistent with artisanal-type salami. Despite this, only limited growth of white mold was observed during the process. All the salami samples were manufactured without the addition of nitrite/nitrate salts.

### 2.2. Sampling and Physicochemical Analyses

Three slices (15 mm thickness) were isolated from each salami sample (one from each end and one from the center), and after the casings were removed, they were coarsely cut by hand knife and immediately finely ground using a knife mill (three times at 2500 rpm × 10 s, Grindomix Retsch, Dusseldorf, Germany). The sample homogenate was used for analyses of pH, Thiobarbituric Acid Reactive Substances (TBARs), Non-Protein Nitrogen (NPN), heme iron, alpha-tocopherol, and NaCl content, as well as for fat extraction. For fat determination, an aliquot of finely ground sample (10 g) was homogenized (13,000 rpm min^−1^ × 60 s with a disperser UltraTurrax T25 basic Ika Werke, Staufen, Germany) with 170 mL of a binary mixture of chloroform/methanol solvents (2/1 *v*/*v*). The extract was washed with 34 mL of a saline solution (NaCl 0.58%). After centrifugation at 3000 rpm for 20 min at 4 °C (Avanti J-E Beckman Coulter centrifuge, Brea, CA, USA), the supernatant fraction was discarded, and the remaining organic fraction was filtered (paper filter, Whatman 1PS, Little Chalfont, Buckinghamshire, UK), dehydrated (sodium sulphate anhydrous, Sigma-Aldrich, St. Louis, MO, USA) and brought to dryness in a rotary evaporator (Buchi Rotavapor, R-215, Flawil, Switzerland) [40]. Dehydrated fat was used for diene number, titratable acidity and fatty acid profile determinations.

The pH was measured after sample homogenization (9000 rpm min^−1^ × 30 s with a disperser UltraTurrax T25 basic Ika Werke, Staufen, Germany) in distilled water (1:10, *w*/*v*) using a Portamess pH-meter (Knick 910, Berlin, Germany) equipped with INLAB 427 electrode (Mettler Toledo, Urdof, Switzerland).

For water activity (a_w_) determination (hygrometer AquaLab 4 TEV; Decagon Devices, Pullman, WA, USA), a salami slice (2 mm thickness), trimmed of the ring immediately below the casing, was roughly chopped and placed in a Teflon cup for direct measurement.

Primary lipid oxidation was measured as conjugated dienes (CD) by dissolving 0.1 g of dry fat in 25 mL cyclohexane (spectrophotometric grade) and measuring UV absorbance at 233 nm using a Jasco V-650 spectrophotometer (JASCO International Co., Ltd., Tokyo, Japan). Since triglycerides exhibit strong absorption at 200–225 nm, which overlaps with CD absorption, a second-derivative (SD) spectrum was used to resolve the signal from background absorption. Results are expressed as the maximum height between the two minimum values of the SD spectrum [41]. Limit of quantification (LOQ) and limit of detection (LOD) were estimated using conjugated (9E,11E)-Linoleic acid (Sigma-Aldrich) equal to 0.001 and 0.0003 absorbance unit, respectively.

Salami samples were analyzed for secondary lipid oxidation products after the extraction of malondialdehyde (MDA) from 5 g of sample using a 5% trichloroacetic acid solution. The purified extract was reacted with 2-thiobarbituric acid, and the pigment measured spectrophotometrically at 532 nm. The result was indicated as mg MDA/kg sample [42]. NPN was measured according to [43] and expressed as a percentage of the ratio between soluble nitrogen in 12% trichloroacetic acid and total nitrogen calculated by difference [39].

Acidity index (AI) was determined through the titration of free fatty acids (FFA) [44] in 2.5 g of anhydrous fat dissolved in 50 mL of diethyl ether/ethanol 1/1 (*v*/*v*) and titrated with sodium hydroxide 0.1 M using phenolphthalein as an indicator. The results are indicated as grams of oleic acid per 100 g of fat using the molar mass of 282 (g/mol).

Heme iron was determined from 10 g of finely minced sample and expressed as mg/100 g of fresh tissue [45]. This analysis was conducted only on samples at 20 weeks of processing.

For the determination of alpha-tocopherol, an aliquot of 0.5 g of finely ground sample was weighed into a screw-cap tube, and 2.5 mL ethanol and 1 mL of KOH 50% (*w*/*v*) were added. The tube was flushed with nitrogen and incubated in a water bath at 80 °C for 30 min with gentle agitation. After cooling in an ice bath, the mixture was extracted with 10 mL of diethyl ether/petroleum ether (50/50) containing 0.02% BHT, with vigorous mixing for 2 min, twice. Cold water (10 mL) was added, and after shaking for 10 s, the mixture was centrifuged at 1000 rpm for 10 min (centrifuge 5804 R, Eppendorf, Hamburg, Germany). An aliquot of the organic phase (3 mL) was evaporated to dryness under vacuum and re-dissolved in 0.3 mL of methanol, then filtered through a 0.45 μm membrane filter prior to injection into the HPLC system (Waters, Milford, MA, USA) equipped with a Discovery C18 reverse-phase column (150 mm × 4.6 mm, 5 μm, Supelco Inc., Bellefonte, PA, USA) maintained at 25 °C. Chromatographic separation was performed under isocratic conditions with a mobile phase of methanol/water (98/2, *v*/*v*) at a flow rate of 0.8 mL/min and an injection volume of 20 μL. Alpha-tocopherol was detected by UV absorbance at 293 nm. Quantification was performed using an internal standard, with a known amount of ergocalciferol added to the sample after saponification, and the resulting signal was detected at 264 nm [46,47]. The technical performance of the analytical protocol has been validated according to [48].

The NaCl content was determined for 10 g of finely minced sample using the Volhard method [49].

For the FA profile, 40 mg of anhydrous fat was transesterified [50]. Fatty acid methyl esters (FAME) were analyzed using a 2010 Plus automated gas chromatograph (Shimadzu Italia, Milano, Italy) equipped with an Omegawax 250 capillary column (30 m × 0.25 mm × 0.25 μm) (Sigma-Aldrich, St. Louis, MO, USA) and a flame ionization detector. Helium was used as the carrier gas at a constant flow rate of 0.8 mL min^−1^, whereas both injector and detector were set at 255 °C. The oven temperature program was as follows: an initial temperature of 80 °C held for 2 min, ramped to 255 °C at 3.5 °C min^−1^, then held for 15 min. The FAs were identified by comparing their retention times to those of authentic FAMEs using a 37-Component FAME mix standard (Supelco, Bellefonte, PA, USA). Results were expressed as a relative percentage of total FAME and as g/100 g of salami.

Moisture:protein (M:P) was calculated as the ratio between moisture percentage and protein percentage, whereas water phase salt (WPS) was calculated as the percentage ratio between salt percentage and the sum of salt and moisture percentage [39].

### 2.3. Sensory Evaluation

At the end of each ripening process (10 and 20 weeks), sensory analysis was conducted over 6 days. Each day of analysis, a total of 8 salami samples (*n* = 1/treatment) were removed from the ripening chamber, packed, and transported to the Laboratory LabCNX of the Department of Animal Medicine, Production and Health (MAPS-University of Padova) for sensory evaluation. The panel consisted of six trained panelists from the Department’s staff. Therefore, for each ripening time, a total of 6 salami samples per treatment were evaluated by all panelists. Panelists had previous experience on the tested matrix (salami sensory evaluation). Moreover, prior to the formal sensory sessions, the panel participated in two training sessions aimed at choosing the appropriate lexicon/descriptors for the tested product, including harmonizing the interpretation of each descriptor. The sensory evaluation was based on a continuous line scale with numerical intensity references from 1 to 9 for each examined descriptor. The physical length of the scale was 15 cm. The left end represented the lowest intensity, and the right end the highest intensity. The position marked by each panelist was measured in millimeters from the left endpoint and used as the sensory score.

The first phase of the sensory evaluation focused on the product’s external appearance. Salami samples were presented on a table with random 3-digit number codes; panelists were asked to evaluate and rate shape (not regular or regular), casing uniformity (not uniformly linked to the salami or uniformly linked), and mold presence (none or high). Subsequently, each panelist moved to their workstation, which was provided with still water at room temperature and unsalted crackers. Each salami sample was sliced by a person who was not part of the panel, and two slices per sample were provided to each panelist. Panelists were first asked to evaluate the appearance of each slice based on the following descriptors: color homogeneity (low–high), cohesiveness (low–high), and fat inclusion (low–high). Afterwards, each panelist was asked to sniff the slice and evaluate its odor. The following attributes were scored: intensity, moldy, rancidity, gamy, and spicy (low–high). The flavor analysis included gamy, salty, rancidity, metallic, fatness, moldy, spicy and flavor persistency (low–high). Thereafter, the panelists evaluated the intensity of off-flavors (low–high), texture attributes including tenderness and juiciness (low–high), and perception of the degree of maturity (too fresh–too ripe).

### 2.4. Statistical Analysis

Data were analyzed using SAS 9.1.3 statistical analysis software for Windows (2004) through General Linear Model (GLM) procedures. A three-way ANOVA was performed, stratified by ripening time (10 and 20 weeks), with tested fat, salt and LAB starter cultures as fixed effects on pH, a_w_, CD, TBARs, NPN, AI, heme iron, alpha-tocopherol, NaCl, M:P and WPS. The statistical analysis also considered the following interactions: fat × salt; fat × lab; salt × lab; and fat × salt × lab, where the lab acronym is the starter culture). When no significant interactions were found, only main effects were considered. Least square means were obtained using the Bonferroni test. Because only one manufacturing batch was produced for each treatment combination, batch and treatment were completely confounded, and the between-batch variance could not be estimated separately. Therefore, statistical inference should be interpreted as referring to the salami produced in the present experimental manufacturing trial, and extrapolation to a wider population of production batches should be made with caution. Model assumptions were evaluated by graphical inspection of the residuals (normal probability plots and residual-versus-predicted plots). No evidence of substantial deviations from normality or homogeneity of variance was observed. Although sensory scores were obtained using a bounded intensity scale, the residual diagnostics did not reveal departures from the assumptions required for the application of the GLM, consistent with the widespread use of parametric ANOVA for trained-panel sensory data.

Correlations between variables were determined using Pearson’s linear correlation coefficient (IBM SPSS Statistics 29.0.1.0). Principal component analysis (PCA) was performed to investigate the relationships among ostrich salami with different fat levels (30% FAT30 and 40% FAT40), NaCl (2.4% and 2.6%), and starter culture mixtures (LAB6: *Lactobacillus curvatus*/*Staphylococcus xylosus* + dextrose; LAB8: *Lactobacillus sakei*/*Staphylococcus xylosus* + dextrose) at 10 and 20 weeks of ripening, considering physicochemical analyses, lipid oxidation, acidity index, proteolysis index, alpha-tocopherol content, FA composition, and sensory properties. Differences were considered statistically significant at *p* < 0.05.

## 3. Results

### 3.1. Effects of Fat, Salt and Starter Cultures

#### 3.1.1. Physicochemical Parameters

The chemical parameters of ostrich salami manufactured using two levels of fat inclusion (FAT30 vs. FAT40), salt inclusion (2.4% vs. 2.6%) and starter cultures (LAB6: *Lb. curvatus*/*S. xylosus* vs. LAB8: *Lb. sakei*/*S. xylosus*), are presented in Table 1 (10 weeks of ripening) and Table 2 (20 weeks of ripening). Overall, the tested treatments affected almost all chemical variables at both time points. Also, at 10 weeks of ripening, several interactions were observed, whereas very few were observed at 20 weeks of ripening.

After 10 weeks of ripening, salami with different fat and NaCl levels exhibited different pH values: a higher pH was recorded in salami with lower fat content and reduced salt addition (*p* < 0.05). However, pH did not differ between the starter culture groups (LAB 6 vs. LAB 8). After 20 weeks of ripening, the pH differences associated with fat content became even more pronounced, indicating a greater tendency for pH to increase in salami with the lowest fat inclusion. In addition, LAB6 produced a lower pH than LAB8. At this point, a significant fat × lab interaction was also depicted: in salami containing 30% fat, the pH value was the same (5.6) for both starter cultures (*p* > 0.05), whereas in salami manufactured with 40% fat, the pH decreased from 5.4 to 5.3 in LAB8 and LAB6, respectively (*p* < 0.001).

Water activity (a_w_) was also affected by the treatments, at both 10 and 20 weeks of ripening. After 10 weeks of ripening, the a_w_ was lower in salami containing 30% fat (*p* < 0.001), 2.6% NaCl (*p* < 0.001), and inoculated with LAB6 (*p* < 0.001); also, a significant three-way interaction among fat content, salt concentration, and starter culture was observed (*p* < 0.001). After 20 weeks of ripening, the difference in a_w_ between salami samples with 30% and 40% fat became even more pronounced (*p* < 0.001). In addition, a significant effect of salt concentration was observed, but this effect was no longer evident when accounting for the starter culture factor.

At 10 weeks of ripening, salt concentration in salami was affected by fat content (*p* < 0.001), with salami containing 30% fat having a higher salt concentration than salami containing 40% fat (3.73% vs. 3.21%). As expected, salami manufactured with 2.6% NaCl also showed higher (*p* < 0.001) salt values than salami made with 2.4% salt inclusion (3.69% vs. 3.25%, respectively). Moreover, batters inoculated with LAB8 exhibited higher (*p* < 0.01) salt concentrations than those prepared with LAB6 (3.56% vs. 3.39%). The significant fat × salt interaction showed that in the 30% fat group, the salt percentage increased from 3.4% to 4.0% (*p* < 0.001), while in the 40% fat group, it increased from 3.1% to 3.4% (*p* < 0.05) for 2.4% and 2.6% salt inclusion levels, respectively. After 20 weeks of ripening, the results observed at 10 weeks were largely confirmed, except for the starter culture effect, as it did not affect any of the traits considered, including salt concentration.

The M:P indicates the level of dehydration in the lean fraction of the salami; the treatments affected this trait at both 10 and 20 weeks of ripening. At 10 weeks, significant differences were observed in salami manufactured with different fat percentages (1.29 vs. 1.75; *p* < 0.001), salt inclusion levels (1.54 vs. 1.50; *p* < 0.05), and starter cultures (1.49 vs. 1.55; *p* < 0.001). The fat × salt interaction (*p* < 0.001) showed that in 30% fat salami, the M:P in salami formulated with 2.6% salt was higher than in samples formulated with 2.4% salt (1.33 vs. 1.26 for 2.6% and 2.4% salt inclusion levels, respectively; *p* < 0.001). However, in salami manufactured with 40% fat, the opposite was observed (1.68 vs. 1.83 for 2.6% and 2.4% salt inclusion, respectively; *p* < 0.001). The fat × lab interaction highlighted that, in salami having 30% fat, the M:P was equal to 1.30 and 1.28 in LAB6 and LAB8, respectively (*p* > 0.05), while in salami with 40% fat, the M:P was 1.68 and 1.72 (for LAB6 and LAB8, respectively, *p* < 0.001). After 20 weeks of ripening, the only significant effect observed for the M:P was the fat inclusion level: 0.96 vs. 1.45 for 30% and 40% fat, respectively (*p* < 0.001).

At 10 weeks of ripening, the WPS, which expresses the salt concentration in the aqueous phase of the product (important indicator of microbiological risk), differed depending on the fat (10.1% vs. 8.1% for 30% and 40% fat, respectively; *p* < 0.001) and salt (8.6% vs. 9.6% for 2.4% and 2.6% salt, respectively; *p* < 0.05) inclusion levels. However, at 20 weeks of ripening, only the fat inclusion level affected the WPS (14.8% vs. 11.5% for 30% and 40% fat; *p* < 0.001). The alpha-tocopherol content varied with fat inclusion level at both 10 and 20 weeks of ripening, with 30% fat salami showing higher values than 40% fat salami (*p* < 0.001). Regarding heme iron content, which was analyzed only at 20 weeks of ripening, a higher amount was observed in salami manufactured with 30% fat compared to samples containing 40% fat (12.4 vs. 8.8 mg/100 g for 30% and 40% fat, respectively; *p* < 0.001).

#### 3.1.2. Lipolysis and Lipid Oxidation

The acidity index is a synthetic indicator that expresses the percentage of hydrolyzed fat, i.e., free fatty acids (FFA). This parameter was affected by the treatments at 10 weeks of ripening, but not at 20 weeks (Table 1 and Table 2). Salami manufactured with 30% fat, 2.4% salt, and LAB6 inoculation had higher values than samples formulated with 40% fat (*p* < 0.001), 2.6% salt (*p* < 0.001), and LAB8 (*p* < 0.05). A significant fat × salt interaction was also observed: in 30% fat salami, FFA increased from 8.94 to 9.23 g of oleic acid/100 g of fat (*p >* 0.05), while in 40% fat salami, FFA increased from 7 to 8 g of oleic acid/100 g of fat (*p* < 0.05) for 2.6% and 2.4% salt, respectively.

The content of CD was influenced by the treatments at 10 weeks of ripening (Table 1), whereas at 20 weeks, it remained unaffected (Table 2). Results indicated that CD were the highest in salami samples with the 40% fat inclusion (*p* < 0.001), in those manufactured with 2.4% salt (*p* < 0.001), and in those inoculated with LAB8 (*p* < 0.001), respectively. The fat × lab interaction (*p* < 0.001) indicated that, at 40% fat inclusion, LAB6 and LAB8 showed comparable outcomes, whereas at 30% fat, LAB8 displayed the highest CD content. The fat × salt interaction (*p* < 0.001) explained that CD were lower in salami samples with 2.6% salt than in those with 2.4% salt, both with 40% fat (0.007 vs. 0.01 for 2.6% and 2.4% salt, respectively) and 30% fat (0.004 vs. 0.009 for 2.6% and 2.4% salt, respectively) inclusions. The significant salt × lab interaction (*p* < 0.001) showed that in salami prepared with 2.4% salt, the CD values increased from 0.006 (LAB6) to 0.013 (LAB8). The opposite was observed in salami formulated with 2.6% salt: the CD values decreased from 0.006 (LAB6) to 0.004 (LAB8).

Results regarding the secondary products of lipoperoxidation (TBARs) indicated that only the fat inclusion level exerted a significant effect, both at 10 (*p* < 0.001) and 20 (*p* < 0.05) weeks of ripening (Table 1 and Table 2): salami with 30% fat displayed higher values than salami belonging to the 40% group (10 weeks: 0.36 vs. 0.21 mg/kg, 20 weeks: 0.34 vs. 0.22 mg/kg). At 10 weeks, a significant salt × lab interaction (*p* < 0.001) was observed: in 2.4% salt salami, TBARs values increased from 0.16 to 0.40 mg/kg (LAB6 vs. LAB8, respectively), while in 2.6% salt salami, TBARs were higher in LAB6 than in LAB8 (0.40 vs. 0.18, respectively).

#### 3.1.3. Proteolysis

After 10 weeks of ripening (Table 1), salami manufactured with 40% fat showed a higher percentage of non-protein nitrogen (NPN) than that with 30% fat (18.4 vs. 15.1%; *p* < 0.001). Also, salami inoculated with LAB8 evidenced a higher NPN than samples prepared using LAB6 (17.3 vs. 16.2%; *p* < 0.001). At 20 weeks of ripening (Table 2), a similar result was observed for NPN relative to the fat inclusion level: 16.9 vs. 14.9% for FAT40 and FAT30, respectively (*p* < 0.01).

#### 3.1.4. Fatty Acids

The fatty acid (FA) profile is presented as relative percentage of fatty acid over total FAME (Table 3 and Table 4) and as absolute content in g/100 g salami (Appendix A, Table A1 and Table A2). The major differences in the fatty acid composition in the present study after 10 weeks of ripening were mainly a function of the percentage of fat inclusion, for which a higher concentration of saturated FA (SFA) (39.8% vs. 38.8%; *p* < 0.0001) and polyunsaturated FA (PUFA) (11.7% vs. 11.2%; *p* = 0.0199) was observed in the FAT30 group, while monounsaturated FA (MUFA) were higher in the FAT40 group (47.2% vs. 45.8%; *p* < 0.0001). Similarly, the sum of *n*-6 and *n*-3 was significantly higher in the FAT30 salami, while the *n*-6/*n*-3 ratio was lower (19.0 vs. 20.1; *p* = 0.0488). Regarding the fat quality indices, the atherogenic index (AI), thrombogenic index (TI), and hypocholesterolemic:hypercholesterolemic ratio (hH) were found to be more advantageous (*p* < 0.0001) in the salami with the highest fat concentration (Table 3). Starter cultures affected the SFA proportion, with LAB6 showing a higher SFA percentage than LAB8 (*p* = 0.008).

After 20 weeks of ripening, only PUFA, total *n*-6, and AI did not differ between the two fat levels (Table 4), whereas the starter culture effect became irrelevant. According to absolute quantitative data, at 10 weeks of ripening, the FA levels in ostrich salami were partly affected by the fat inclusion percentage and the starter cultures, whereas the salt inclusion percentage did not exert any influence (Appendix A, Table A1). A greater inclusion of fat (40%) into the salami batter determined higher SFA (13.6 vs. 12.8 g/100 g salami for FAT40 and FAT30, respectively; *p* < 0.0001) and MUFA (16.5 vs. 14.7 g/100 g salami for FAT40 and FAT30, respectively; *p* < 0.0001), but did not modify total *n*-3, *n*-6 and overall PUFA (*p* > 0.05). Salami inoculated with LAB6 led to higher amounts of SFA (13.6 vs. 12.7 g/100 g salami for LAB6 and LAB8, respectively; *p* < 0.0001) and MUFA (16.1 vs. 15.1 g/100 g salami for LAB6 and LAB8, respectively; *p* = 0.0001) than with LAB8. Also, in this case, PUFA were not affected by the starter cultures. At 20 weeks of ripening, the effects observed at 10 weeks were not confirmed, as none of the tested treatments affected the FA levels in salami (Appendix A, Table A2).

#### 3.1.5. Sensory Characteristics

Results of the sensory analysis of ostrich salami conducted after 10 weeks of ripening (Table 5) highlighted notable effects depending on the fat inclusion level, salt percentage, and, to a minor extent, the starter culture. Overall, the level of fat inclusion influenced almost all sensory descriptors (visual, olfactory, gustative, and textural): salami manufactured with 40% pork fat showed a more regular shape, greater casing adhesion, and more evidence of fat. On the other hand, mold growth and cohesion between fat and lean meat were greater in the FAT30 salami than in the FAT40. FAT40 salami had higher gamy (*p* < 0.001) and lower spicy (*p* < 0.001) odors compared to FAT30. Also, FAT40 salami had higher off-flavors intensity than FAT30 (*p* < 0.001): gamy (*p* < 0.001), metallic (*p* < 0.01), fatness (*p* < 0.001), and moldy (*p* < 0.001) attributes were higher in the 40% fat salami, while the salty flavor was higher in the 30% fat salami (*p* < 0.001). FAT40 salami also displayed a greater tenderness (*p* < 0.001) and juiciness (*p* < 0.001) than FAT30.

Salami manufactured with a higher salt inclusion (2.6%) had the greatest color homogeneity (*p* < 0.001), the highest overall odor (*p* < 0.001), and rancid odor (*p* < 0.001) intensities. However, the metallic (*p* < 0.01) and moldy (*p* < 0.01) flavor attributes received higher scores in 2.4% salt salami than in 2.6%. Unlike the fat inclusion level, different salt percentages in manufactured ostrich salami did not influence off-flavor intensity and textural attributes. Different starter cultures influenced the external appearance, odor characteristics, and off-flavors of ostrich salami. LAB8 salami samples were characterized by a higher shape regularity (*p* < 0.01) and casing adhesion (*p* < 0.001) than LAB6. Also, LAB8 enhanced overall odor (*p* = 0.05) and moldy (*p* < 0.05) odors compared to LAB6, whereas the latter group was characterized by a less intense rancid odor (*p* < 0.05) compared to LAB8. Also, the intensity of off-flavors was higher in LAB8 compared to LAB6 salami (*p* < 0.001). The intensity of different flavor attributes was superior in the FAT40 salami and in the salami with the LAB6 starter, for which a significant interaction was also observed (fat × lab; *p* < 0.001). Gamy flavor intensity in LAB6 did not change, increasing only slightly from 2.6 to 2.8 (*p* > 0.05) for FAT30 and FAT40, respectively, whereas in LAB8, it increased from 2.1 to 3.1 (*p* < 0.001) for FAT30 and FAT40, respectively. The intensity of moldy flavor in LAB6 increased from 1.3 to 2.8 (*p* = 0.002) for FAT30 and FAT40, respectively, while in LAB8, it rose from 1.5 to 1.8 (*p* > 0.05) for FAT30 and FAT40, respectively. Therefore, an increase in the intensity of some flavor attributes in FAT40 was observed, but this effect varied according to the type of starter culture. In the case of moldy flavor, the higher intensity found for LAB6 (*Lb. curvatus*) was unrelated to mold growth on the salami surface itself, but was more likely directly or indirectly related to the synthesis of key mold-related compounds (1-octen-3-ol, methyl ketones, and methyl aldehydes). After 20 weeks of ripening (Table 6), SALT and LAB treatments did not influence the tested sensory attributes of ostrich salami, whereas FAT remained an influential factor, although to a lesser extent compared to the results observed after 10 weeks of ripening.

### 3.2. Effect of Ripening Period

#### 3.2.1. Physicochemical, Lipid Oxidation, Proteolysis, and Fatty Acid Indicators

Ripening time had a significant effect on various physicochemical attributes, including lipid oxidation, proteolysis, and FA traits measured in ostrich salami (Table 7). A longer ripening duration led to an increase in the amount of salami free FA (FFA) (*p* < 0.001), salt concentration (*p* < 0.001), WPS (*p* < 0.001), and overall PUFA percentage (*p* < 0.001), the latter attributable to both *n*-6 (*p* < 0.001) and *n*-3 (*p* < 0.001) classes. Consequently, an augmented PUFA proportion led to a greater ratio to SFA (*p* < 0.001). There were also variables that decreased as a function of the ripening duration: NPN expressed on the DM (*p* < 0.001), a_w_ (*p* < 0.001), M:P (*p* < 0.001), as well as total SFA (*p* < 0.001) and MUFA (*p* < 0.05) proportions (% of total FAME; Table 7). The above-mentioned variations (increase and decrease as a function of the ripening duration) in the FA profile of ostrich salami led to a reduction in the MUFA:SFA (*p* < 0.001) and SFA:UFA (*p* < 0.001) ratios.

#### 3.2.2. Sensory Characteristics

**Figure 1:**
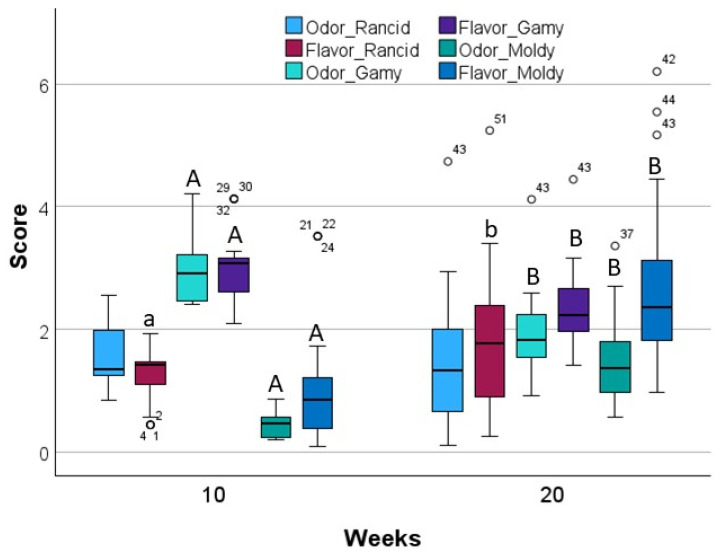
Effect of ripening time (10 vs. 20 weeks) on the sensory attributes Odor rancid, Flavor rancid, Odor gamy, Flavor gamy, Odor moldy and Flavor moldy of ostrich salami. Different superscript capital letters (A, B) within each attribute indicate significant differences for *p* < 0.001; different superscript lowercase letters (a, b) within each attribute indicate significant differences for *p* < 0.05.

**Figure 2:**
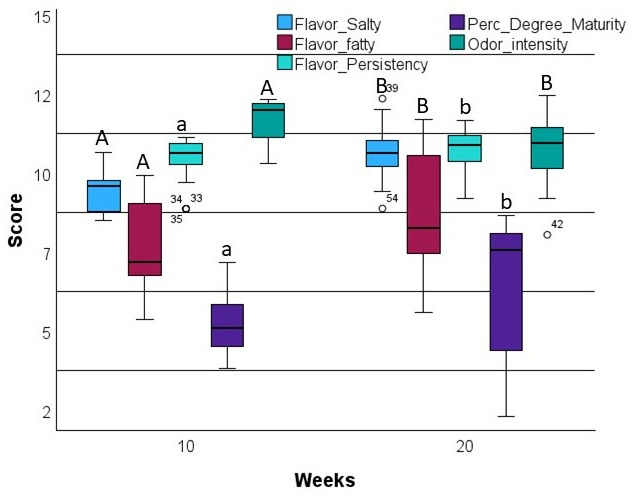
Effect of ripening time (10 vs. 20 weeks) on the sensory attributes of Flavor salty, Flavor fatty, Flavor persistency, Ripening length and Odor intensity of ostrich salami. Different superscript capital letters (A, B) within each attribute indicate significant differences for *p* < 0.001; different superscript lowercase letters (a, b) within each attribute indicate significant differences for *p* < 0.05.

**Figure 3:**
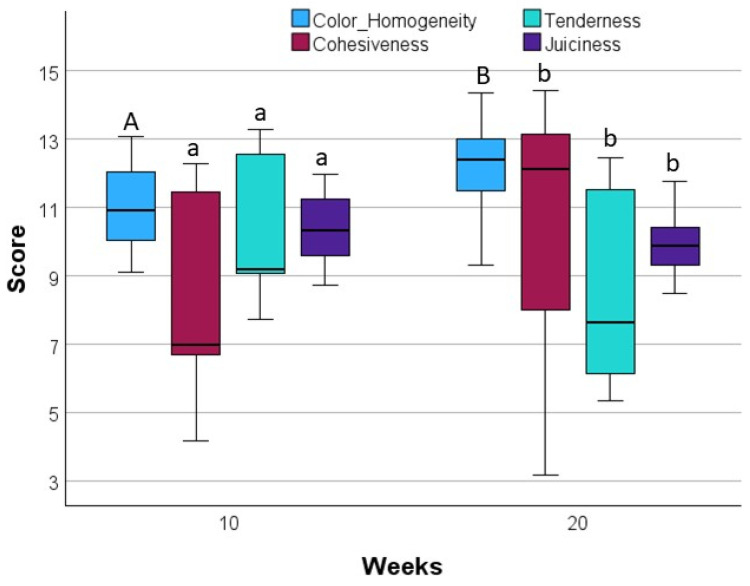
Effect of ripening time (10 vs. 20 weeks) on the sensory attributes of Color homogeneity, Cohesiveness, Tenderness and Juiciness of ostrich salami. Different superscript capital letters (A, B) within each attribute indicate significant differences for *p* < 0.001; different superscript lowercase letters (a, b) within each attribute indicate significant differences for *p* < 0.05.

The influence of ripening duration on the sensory attributes of ostrich salami is shown in Figure 1 and Figure 2. In Figure 1, rancid odor was not affected by ripening duration, whereas rancid flavor perception increased in the period from 10 to 20 weeks (*p* < 0.01). A similar trend was observed for moldy odor (*p* < 0.05) and flavor (*p* < 0.05). Conversely, gamy odor (*p* < 0.05) and flavor (*p* < 0.05) decreased with increasing ripening duration. Figure 2 shows that the flavor intensity of salty (*p* < 0.001), fatty (*p* < 0.001), and persistency (*p* < 0.05), as well as the perception of the degree of maturity (intended as the estimation of the characteristic ripening flavor of fermented dry sausage, *p* < 0.05), increased, whereas the overall odor intensity (*p* < 0.001) decreased in the period from 10 to 20 weeks of ripening. Figure 3 depicts color homogeneity, cohesiveness, tenderness, and juiciness. All were influenced by ripening duration, with color homogeneity (*p* < 0.001) and cohesiveness (*p* < 0.05) increasing. In contrast, tenderness (*p* < 0.05) and juiciness (*p* < 0.05) were negatively affected by ripening duration.

## 4. Discussion

### 4.1. Effects of Fat, Salt and Starter Cultures

#### 4.1.1. Physicochemical Parameters

In the present manufacturing trial, FAT30 salami exhibited higher pH values than FAT40 salami, a result consistent with previous findings. Gómez and Lorenzo [52] reported similar results for “Chorizo” prepared at three levels of subcutaneous pork fat (10%, 20%, and 30%). Comparable trends in two studies were also reported in “Salchichón” formulated with three different levels of pork back fat (10%, 20%, and 30% [53] and 20%, 25%, and 30% [54]). In the aforementioned studies, pork meat was used as the lean component. Similar results were obtained in “Pitina”, a typical fermented meat product formulated with two different percentages of pork lard (10% and 30%) and sheep meat for the lean part [55]. In contrast, no significant differences in pH were observed at the end of the ripening process among three different batches of salami prepared with three different percentages of pork back fat (10%, 20%, and 30%) using pork as lean meat [12]. The difference in pH values as a function of fat percentage became more pronounced at 20 weeks of ripening, likely due to the progressive accumulation of soluble nitrogenous compounds derived from protein hydrolysis, including peptides, free amino acids, ammonia, and biogenic amines, all of which exert a buffering effect on the product matrix [56]. The pH values recorded at 20 weeks of ripening were consistent with those reported in “Chorizo” and “Salchichón” prepared with ostrich meat [37].

Fat interferes with salt diffusion in the mixture, reducing the movement of water toward areas of greater osmotic strength. The combined effect of dehydration and salt diffusion was particularly evident in the low-fat salami, where a higher salt concentration was observed (3.73% vs. 3.21%). This, together with the greater weight loss during ripening [39], was associated with a more pronounced reduction in a_w_ at both 10 and 20 weeks of ripening in the salami evaluated in the present trial. This was consistent with previous research [54,55,57]. However, others found no significant differences in the final a_w_ as a function of the fat percentage of the batter [11,12,58,59], while lower final a_w_ values in “Chorizo” with the higher percentage of added fat were noted, probably due to the significantly lower pH of this batch that greatly reduces the water-holding capacity of the meat proteins [52].

The starter culture *Lb. curvatus* had a significant effect on a_w_, with significantly lower values at 10 weeks compared to salami inoculated with *Lb. sakei* [60], whereas [61] did not observe any significant effect on a_w_ from a strain of *Lb. curvatus* inoculated into sausage batter during fermentation. At 10 and 20 weeks of ripening, a_w_ was significantly correlated with salt concentration (r = −0.658, *p* < 0.001 and r = −0.706, *p* < 0.001, respectively). The M:P provides information on the extent of drying of the lean meat portion, whereas the WPS percentage measures the salt concentration specifically within the water phase of the product. Both parameters are useful for estimating shelf life and safety of meat products, as they are correlated with a_w_ (r = 0.900; *p* < 0.001 and r = −0.906; *p* < 0.001 for a_w_ to M:P and WPS, respectively). Final values of 0.90 to 0.91 for a_w_ and 2:1 for the M:P can be considered as the borderline defining dry and semidry-fermented sausages. At 10 weeks of ripening, M:P values < 1.9, pH < 5.8, and a_w_ < 0.90 are technical characteristics of a long ripening and drying process, which renders the product stable at room temperature and therefore allows it to be classified as dry sausage [27]. From a shelf-stability perspective, the pH and WPS values observed in the salami produced during the present manufacturing trial were consistent with physicochemical conditions generally considered adequate for the storage of semi-preserved meat products, including under conditions of temporary temperature abuse [62]. However, as microbiological enumeration was not performed in this study, this inference is limited to the physicochemical characteristics measured and should not be interpreted as direct evidence of microbiological safety. Antimicrobial action occurs in the aqueous phase of the mixture, highlighting the importance of measuring salt concentration in the aqueous fraction (WPS) or, alternatively, a_w_. In the present study, the calculated WPS percentage ranged from 7.2% to 11.3% after 10 weeks and from 9.6% to 17.5% after 20 weeks of ripening. From a “clean label” perspective, the omission of nitrate and nitrite salts is generally recognized as increasing the risk of proliferation of spoilage bacteria and, more specifically from a safety standpoint, *C. botulinum*. To control this risk, it is therefore necessary to correctly and appropriately apply processing technology—during the fermentation step, ensuring a significant reduction in pH in the first few days and a rapid decrease in water activity by competently managing temperature, relative humidity, and air circulation in the processing rooms—as well as to use a starter culture that competes with pathogenic bacteria (e.g., *Lb. sakei*). This should be accompanied by the addition of sugar to the dough to promote lactic acid microflora [63].

Salt inhibits the growth of many spoilage and pathogenic bacterial species, as well as yeasts and molds, depending on the species and the a_w_ of the medium. Gram-positive bacteria are generally more salt-tolerant than Gram-negative bacteria. Most spoilage bacteria grow at a_w_ > 0.9; among pathogens, *Staphylococcus aureus* is best adapted to low a_w_ values (0.83–0.86). In the European Union countries, the critical a_w_ value below which ready-to-eat foods are not a substrate for the growth of *Listeria monocytogenes* is set at 0.92 [64].

#### 4.1.2. Lipolysis and Lipid Oxidation

FFA are produced through the action of tissue and microbial lipases and phospholipases that hydrolyze triglycerides and phospholipids. In the present study, the effect of fat, salt, and starter cultures on FFA concentration was observed only after 10 weeks of ripening. FFA levels were higher in salami with 30% fat inclusion, which exhibited a significantly lower fat and moisture content and a higher protein concentration compared to salami with 40% fat [39]. In muscle tissue, membrane lipids represent approximately 30% of total fat, while intramuscular fat constitutes 1.2% of total fat [65]. Therefore, the contribution of phospholipids to the overall FFA concentration is expected to be greater in FAT30 than in FAT40 salami. This finding is somewhat unexpected, as several studies have reported a positive relationship between fat content and FFA concentration. Franco et al. [66] reported that the FFA concentration increased with total fat concentration in four different types of dry-fermented sausage produced in Galicia, Spain. Similarly, Olivares et al. [67] observed a proportional increase in FFA concentration with increasing levels of subcutaneous pork fat (10%, 20%, and 30%) in the batter formulation, a trend also noted by others [68,69], who found that the largest proportion of FFA originates from subcutaneous fat triglycerides. In contrast, Chen et al. [70] studied the lean and fat fractions of dry-fermented sausage separately during fermentation, observing that FFA concentration in the lean fraction was higher than in the fat fraction. Ikonić et al. [56] also studied three types of Serbian dry-fermented sausage with fat concentrations of 14.6%, 21.6%, and 38.7%, reporting FFA concentrations of 19.8, 22.4, and 15.2 mg KOH/g fat, respectively, whereas in dry-cured hams, phospholipids are the main substrates for lipolysis [71,72], while triglycerides (TAG) provide a significant amount of FFA in muscles with very high TAG content [71]. Comparing Spanish ostrich “Salchichón” made with ostrich meat plus pork ham with that made purely with ostrich meat, higher FFA were found in the latter one, indicating a stronger lipolytic action in ostrich lean meat [73]. Both pH and a_w_ are intrinsic factors that modulate enzymatic activity and, consequently, FFA concentration. Karshoğlu et al. [74] observed that in fermented salami produced with turkey meat, batches with the lowest pH exhibited the lowest FFA concentration.

In the present study, a significant correlation was observed between both pH and FFA concentration (r = 0.66; *p* < 0.001) and between a_w_ and FFA (r = −0.641; *p* < 0.001). Salt appears to exert a depressing effect on FFA accumulation, with concentrations of 7.97 vs. 8.62 g of oleic acid/100 g of fat observed for 2.6% and 2.4% salt, respectively (*p* = 0.0007). Stahnke [75] observed a reduction in FFA concentration in dry sausage at higher salt concentrations. In contrast, Zhao et al. [76] reported higher FFA concentrations in salami with a higher salt content (4% vs. 2%). The fat × sait interaction is particularly noteworthy. In FAT30 salami, FFA concentrations ranged from 9.2 to 8.9 g of oleic acid/100 g of fat for 2.4% and 2.6% salt, respectively (*p* > 0.05), whereas in FAT40, concentrations ranged from 8 to 7 g of oleic acid/100 g of fat for 2.4% and 2.6% salt, respectively (*p* = 0.0017). These results suggest that as fat content increases, the depressive effect of salt on FFA accumulation becomes more pronounced.

Regarding the effect of starter cultures, LAB6 salami produced higher FFA concentrations than LAB8. Although *Lb. curvatus* does not possess significant lipolytic activity per se, it has been shown to enhance the hydrolytic action of *S. xylosus* [77]. The effect of the two starter cultures on FFA concentration was subject to interaction with salt. At 2.4% salt, no significant difference was observed between the two starters (8.55% vs. 8.69% for LAB6 and LAB8, respectively; *p* > 0.05), whereas at 2.6% salt, LAB6 showed a significantly higher FFA concentration than LAB8 (8.40 vs. 7.55 g of oleic acid/100 g of fat, respectively). According to Molly et al. [68], 60–80% of the lipolytic activity in dry-fermented sausage is carried out by tissue enzymes, while Kenneally et al. [78] did not observe any differences in FFA concentration between sausages inoculated with lipolytic starters and those inoculated with non-lipolytic starters. In the present study, mean FFA concentrations at 10 and 20 weeks of ripening were 8.20 and 12.9 g of oleic acid/100 g of fat, respectively, which are higher than the mean levels of 1–7% reported in other studies [66], possibly reflecting the specific lipid composition of ostrich meat. FAT30 salami had a higher salt concentration and lower a_w_ than FAT40 salami, factors that may have activated and favored the action of acid lipases in the muscle fraction. However, the same factors had a depressive effect on the activity of neutral and basic lipases in adipose tissue [79]. Considering FFA oxidation is relevant because it represents the subsequent step in lipid degradation, leading to the formation of hydroperoxides and their hydrolysis into secondary compounds such as aldehydes, ketones, organic acids, alcohols, and esters, all of which contribute significantly to the flavor profile of dry-fermented salami [80].

CD concentration is an indicator of hydroperoxide formation and represents an early marker of lipid oxidation. In the present study, at 10 weeks of ripening, a significant effect was observed for all three experimental factors. Salami with the lowest salt inclusion (2.4%) exhibited higher CD concentrations compared to salami with 2.6% NaCl, suggesting that higher NaCl content may exert a protective effect against primary lipid oxidation, possibly through inhibition of pro-oxidant enzyme activity. This result contrasts with the findings of [76], who reported higher hydroperoxide concentrations in salami with the highest NaCl inclusion; however, it should be noted that their observations were limited to the first processing step (fermentation phase), which may account for the discrepancy.

Regarding the effect of the starter cultures, LAB6 was associated with a lower CD concentration than LAB8; however, this effect was significantly influenced by the interaction with fat percentage. Specifically, in salami FAT40, no significant difference was observed between the two starter cultures, whereas in salami FAT30, the CD concentration was significantly higher in LAB8 than in LAB6 (0.009 vs. 0.004, respectively; *p* < 0.0001). This finding may be attributed to the antioxidant properties of LAB, which act through free radical scavenging and metal chelation, particularly with metals involved in the Fenton reaction (Cu and Fe) [81,82]. *Lb. curvatus* (LAB6) appears to possess a higher antioxidant capacity than *Lb. sakei* (LAB8) [82,83], which may account for the lower CD concentration observed in LAB6-inoculated salami. Therefore, an apparent paradox arises: FAT30 salami at 10 weeks of ripening showed higher FFA, lower CD, and higher TBARs, as did LAB6, which exhibited higher FFA and lower CD than LAB8. It should be kept in mind that CD is not a direct measure of hydroperoxide groups in the PUFA carbon chain, but rather reflects the shift of a double bond as a consequence of radical attack. Moreover, it can be expected that a significant proportion of the FFA in FAT30 consists of PUFA (given the higher PUFA concentration in FAT30 than in FAT40); these, particularly those with three or more double bonds, are more prone to oxidation, and the decomposition of hydroperoxides is catalyzed by the higher heme iron concentration in FAT30, as indicated by the higher TBARs value in this treatment. On the other hand, the potentially higher antioxidant action of *Lb. curvatus* in controlling the primary phase of lipid oxidation warrants further investigation to better understand this effect, also considering that TBARs levels did not differ between LAB6 and LAB8. The apparently contradictory relationships observed between FFA, CD, and TBARs can be explained by considering that lipid oxidation is a dynamic process involving distinct stages of hydroperoxide formation and decomposition. In the case of LAB6 salami, the higher FFA concentration indicates a greater availability of oxidizable substrates. However, the antioxidant activity attributed to *Lb. curvatus* may have limited the initial free-radical reactions responsible for the formation of conjugated dienes. Consequently, substrate availability and primary oxidation are not necessarily directly coupled, as antioxidant mechanisms such as free-radical scavenging and metal chelation may reduce hydroperoxide formation despite a larger pool of FFA. Likewise, the divergent behavior of CD and TBARs observed between FAT40 and FAT30 salami highlights the different kinetics governing primary and secondary lipid oxidation products. The higher CD values in FAT40 suggest a greater accumulation of hydroperoxides during the early stages of oxidation, whereas the higher TBARs values in FAT30 indicate a more extensive decomposition of hydroperoxides into secondary oxidation products such as aldehydes. This interpretation is consistent with the higher concentration of heme iron measured in FAT30 salami, which may have accelerated hydroperoxide breakdown through iron-catalyzed reactions. Therefore, CD reflects the balance between hydroperoxide formation and disappearance at a given time, while TBARs reflects the accumulation of secondary oxidation products, making dissociation between these two indicators possible during the progression of lipid oxidation.

In the present study, the percentage of fat inclusion was the main factor influencing TBARs values, at both 10 and 20 weeks of ripening. Salami FAT30 exhibited the highest TBARs levels, a finding that contrasts with several previous studies [11,52,67], in which TBARs levels increased with higher fat concentrations in the batter. In contrast, Ref. [53] reported higher TBARs in “Salchichón” with a lower fat concentration, while Ref. [55] found no significant differences in TBARs in “Pitina” meat product formulated with 10% or 30% added fat.

It is worth noting that the TBARs values measured in the present study were considerably lower than those reported in other studies and well below the critical threshold of 2 mg MDA/kg, above which rancidity becomes perceptible [84]. Wójciak et al. [85], studying three batches of organically produced fermented sausages and one batch of conventionally produced pork meat (using sodium nitrite) at the end of the ripening period (21 days) and after 90 and 180 days of vacuum storage at cold temperature, reported TBARs values consistently above 1.4 mg MDA/kg, with some samples approaching 3 mg MDA/kg. Similarly, in four batches of pure pork sausages seasoned with acid whey and/or natural antioxidants, vacuum-packed after 21 days of ripening and stored in the refrigerator, TBARs values between 0.92 and 2.35 mg MDA/kg were reported after 90 and 180 days [86]. Olivares et al. [67], using three batches of pure pork mixture with 10%, 20% and 30% subcutaneous fat and sodium nitrite and potassium nitrate in the dressing formulation, reported TBARs values between 1.3 and 1.7 mg MDA/kg dry matter (DM) after 60 days of ripening, which are significantly higher than those measured in the present study, even when expressed on a DM basis (0.54 and 0.33 mg MDA/kg DM for 30% and 40% fat, respectively). In salami obtained from goat meat and pork subcutaneous fat, comparing a control batch without antioxidants with two batches containing antioxidants (rosemary extracts) subjected to 2 weeks of ripening, followed by vacuum-packaging and storage at room temperature for up to 90 days, TBARs values increased during the first weeks and subsequently decreased, reaching final values of approximately 5 mg MDA/kg sample [87].

In the salami of the present study, the significant effect of the percentage of fat inclusion on the residual concentration of alpha-tocopherol was observed both at 10 and 20 weeks of ripening, for which two observations can be made. The first concerns the effect of the ripening period, for which no significant differences in concentration were observed (0.36 vs. 0.40 mg/kg at 10 and 20 weeks, respectively; *p* > 0.05). A minimal but significant antioxidant effect was observed, as indicated by a decrease in CD (r = −0.316; *p* = 0.007), while TBARs levels appeared to be independent of alpha-tocopherol concentration. Alpha-tocopherol is one of the primary antioxidants present in animal tissues; it resides almost solely in phospholipid membranes, of which skeletal muscle is rich due to several different organelles, and among them, mitochondria are the most diffuse due to their metabolic role. In the membranes, the oxidizable substrates are predominantly PUFA, and it has been estimated that as much as 1–2% of all oxygen consumed may result in the formation of reactive oxygen species (ROS), with the vast majority of ROS being generated in the mitochondria. Alpha-tocopherols prevent the propagation of free radicals in membranes, and when peroxyl radicals are formed, these react 1000 times faster with tocopherols than with polyunsaturated fatty acids [88,89]. Sammet et al. [90] observed a significant effect of alpha-tocopherol levels on TBARs values in samples of sliced salami packaged in a protective atmosphere and exposed to light for 12 weeks, together with a significant decrease in the vitamin over the weeks of storage. Harms et al. [91] did not observe any effect of alpha-tocopherol on TBARs levels in sausages aged for 4 weeks and then stored for a further 8 weeks, and, at the same time, did not detect any decrease in alpha-tocopherol concentration during the observation period. A role in the decrease in vitamin E appears to be played by nitrate and nitrite salts added to the batter for preservative purposes. The protective effect of vitamin E against nitrite and nitrate toxicity is attributable to its ability to limit the production and availability of superoxide and NO· [92]. The iron present in the heme proteins appeared to play a significant role in the lipoperoxidation of salami. From a theoretical viewpoint, in animal post mortem processes, among them the pH decline, inactivate the reductive enzyme systems and stimulate acid-catalyzed autoxidation of the iron(II) state to the iron(III) state of myoglobin in meats. Formation of MbFe(III) is highly correlated with the extent of lipid oxidation in muscle foods, and it is generally accepted that MbFe(III) is an effective pro-oxidant at acidic pH and in the presence of hydroperoxides. The iron(II) myoglobin species can likewise react with hydrogen peroxide, whose concentration in dry-fermented sausages is common, resulting in formation of ferrylmyoglobin that is expected to be an effective pro-oxidant under the conditions found in muscle food [93]. In the present study, a higher heme-iron concentration was found in FAT30, where TBARs values were also the highest. In a study on droëwors made using 75% ostrich meat and 25% pork back fat [94], higher concentrations of heme iron and higher levels of TBARs than those observed in the present study were reported.

In conclusion, the higher TBARs values found in FAT30 were the consequence of the higher concentration of PUFA and the concomitantly higher concentration of heme iron, while the antioxidant action of alpha-tocopherol was mainly evident at the initial stage of the oxidative process (lower CD). On the other hand, lipid hydroperoxides were preferentially cleaved to aldehydes and other secondary lipid oxidation products due to an environment that, in FAT30, was richer in iron and probably other pro-oxidant metals than in FAT40.

#### 4.1.3. Proteolysis

The data on fresh weight indicated higher proteolysis in FAT40 salami. Considering the higher DM and protein content of FAT30 (for details, see [39]), when expressed on a DM basis, NPN values were 0.93 and 0.96 g/100 g DM (*p* > 0.05) for FAT30 and FAT40 salami, respectively, at 10 weeks, and 0.89 and 0.84 g/100 g DM (*p* > 0.05) at 20 weeks of ripening. Therefore, the effect of fat inclusion on the proteolysis index was at least partly attributable to differences in protein percentage rather than to actual proteolytic activity. A significant effect of the starter culture type (*p* < 0.0001) and of the fat × lab and salt × lab interactions (*p* < 0.0001) was observed. The greatest accumulation of soluble nitrogen was found in salami inoculated with LAB8 (*Lb. sakei*), particularly in combination with 40% fat (fat × lab interaction) and 2.4% salt (salt × lab interaction). Both *Lb. curvatus* and *Lb. sakei* have demonstrated proteolytic capacity [95,96], although it is difficult to generalize which species is more efficient, as this depends on the strain, experimental conditions, and substrate composition.

The effect of ripening time on proteolysis in fermented salami is well established, with soluble nitrogen accumulation progressively increasing as ripening proceeds [97,98,99]. In the present study, the effect of ripening time was not significant when NPN was expressed on a fresh weight basis; however, DM data suggest that proteolytic activity slowed after 10 weeks of ripening. This deceleration may be attributed to the significant increase in WPS and the concurrent decreases in the M:P ratio and a_w_, which likely reduced proteolytic activity. This is consistent with the known pattern of protease activity in dry-fermented salami, whereby tissue proteases predominate during the early fermentation phase, while microbial proteases become more active during the advanced ripening phase [68].

#### 4.1.4. Fatty Acids

The composition of the fat, in addition to its quantity used in the salami batter formulation, influences the sensory and nutritional properties of the salami as well as its susceptibility to oxidation since FA, and in particular, PUFA, comprise the main substrate for the oxidative process. The major differences in the fatty acid composition in the present study after 10 and 20 weeks of ripening were mainly a function of the percentage of fat inclusion, for which higher concentrations of SFA and PUFA were observed in the 30% fat inclusion, while MUFA was higher in the 40% fat inclusion. Similar results were also observed when comparing the FA profile of dry-fermented sausage produced with lean meat and fat from different pig breeds [94]. The SFA/UFA ratio is an indicator of the consistency of the fat fraction, where an increase in oleic acid results in a decrease in consistency, contrary to what is observed with an increase in stearic acid concentration. A decrease in the SFA/UFA ratio increases the risk of autooxidation phenomena, which are directly proportional to the average level of unsaturation [66]. In this study, the highest level of TBARs was observed in salami with 30% fat, where the concentrations of *n*-6 PUFA and *n*-3 PUFA were higher, while CD was significantly correlated with PUFA concentration (r = −0.538, *p* < 0.001). Ref. [94] made ostrich droëwors whose PUFA percentage in the end-product varied between 15.7% to 18.7%, which caused important lipid oxidation, with TBARs values between 6.7 to 11.3 mg MDA/kg meat. In the present study, the higher percentage of pork back fat employed moved the fatty acid profile of the salami toward lower unsaturation levels (more similar to those of pork than of ostrich meat) with beneficial effects for fat stability during ripening. The PUFA concentration was equal to 11.5% of total FAME at 10 weeks of ripening and increased to 12.2% at 20 weeks. Similar trends were noted also for *n*-6, *n*-3 and the PUFA/SFA ratio, whereas SFA, MUFA, MUFA/PUFA and SFA/UFA ratios decreased in the period from 10 to 20 weeks of ripening. When producing fermented salami intended for long ripening periods, it is advisable to use raw materials with a PUFA percentage not exceeding 12% to avoid the onset of significant fat oxidation and/or structural softening resulting from partial melting of overly unsaturated fat [100]. This observation is consistent with other studies showing that PUFA are predominantly located in the phospholipid fraction of meat, which is more abundant in muscle than in fat storage tissues. After 20 weeks of ripening, the difference in PUFA concentrations attributable to the percentage of fat inclusion was no longer evident.

#### 4.1.5. Sensory Characteristics

The sensory profile of dry-fermented ostrich salami was significantly influenced by fat content, salt concentration, starter cultures, and their interactions, with several differences emerging at the two ripening times. Fat content was the primary factor affecting shape and casing uniformity at 10 weeks of ripening, with 40% fat salami scoring higher than 30% fat salami, consistent with the faster weight loss and surface deformation observed in leaner products [39], as also reported by others [57,101]. These differences disappeared at 20 weeks, suggesting a progressive homogenization of the external appearance during ripening. Ref. [102], in contrast, observed a significantly superior appearance in sausages with an intermediate fat concentration (20%) compared to samples with 10% and 30% fat, while Ref. [103] reported the best evaluation of appearance in salami with the lowest fat concentration at the end of 90 days of ripening. Mold coverage was significantly higher in 30% fat salami at both ripening times, likely due to reduced surface fat exudation in leaner salami, which facilitated fungal colony attachment and growth. A significant fat × lab interaction effect on mold coverage was observed at 10 weeks, indicating a differential effect of starter cultures depending on fat content.

Intrinsic conditions such as high salt concentration and low a_w_, together with lower temperatures in the first weeks of processing, make the surface of salami unsuitable for several mold species. Usually, *Penicillium* spp. represent between 50% and 90% of the fungal species occurring in traditional dry-fermented sausages [104], with the prevalence of *P. nalgiovense*, followed by *P. olsonii* and *P. chrysogenum*, which form a white/whitish-gray/light green layer with a protective effect against some undesirable pathogenic or spoilage microorganisms. Some of the beneficial effects of molds are related to flavor and taste, mediated by lactate oxidation, proteolysis, amino acid degradation, lipolysis, β-oxidation, and color stabilization through catalase activity and oxygen consumption. However, some mold species may represent a serious threat in terms of antibiotic and mycotoxin contamination, potentially causing allergic reactions. Furthermore, homogeneous mold growth also exerts a technological effect by slowing weight loss and contributing to the control of the so-called drying ring. In the present study, proper management of the relative humidity in the ripening chamber limited this defect, as evidenced by the color homogeneity evaluations. Regarding internal appearance, the 30% fat ostrich salami exhibited significantly higher cohesiveness scores at both ripening times, supporting the results of other studies [52,53]. In our study, a significant fat × salt interaction was observed, consistent with the known role of salt in promoting myofibrillar protein extraction and increasing binding capacity [11,57]. Fat inclusion scores were higher in FAT40 ostrich salami, as expected given the greater use of back fat in salami manufacture.

Color homogeneity was significantly influenced by salt concentration at 10 weeks, with higher scores at 2.6% salt, whereas at 20 weeks of ripening, fat content became the dominant factor, with FAT30 salami showing higher scores.

The odor and flavor profiles were predominantly influenced by fat content at both ripening times. In general, higher fat content was associated with more intense gamy, fatty, metallic, rancid, and spicy notes, while leaner salami exhibited more pronounced salty and flavor attributes, but only at 10 weeks of ripening. Regarding odor intensity, no significant effect of fat content was detected at either 10 or 20 weeks of ripening, in contrast with [103], who observed greater odor intensity in higher-fat salami, and [52], who reported the opposite trend. This apparent inconsistency may be explained by the complex relationship between fat content and volatile compound release. Indeed, although instrumental analysis by GC–MS has shown that increasing fat concentration reduces the concentration of volatile compounds in the headspace, particularly the more fat-soluble ones, the sensory perception of odor intensity does not necessarily mirror this pattern. During consumption, volatile compounds are released from the product matrix through chewing and reach the nasal mucosa retronasally, a process that appears to be largely independent of fat concentration [105]. Consequently, the fat content may influence the partition of volatile compounds between the food matrix and the headspace without proportionally affecting their sensory perception.

At 10 weeks of ripening, higher fat content was associated with a greater perception of gamy odor and flavor, with a significant fat × salt interaction suggesting that the combined effect of fat and salt modulates the intensity of this attribute. In lamb and sheep meat, gamy flavor has been strongly and positively correlated with aspartic acid, cyclo-leucine, and gluconic, citric, and pyruvic acid [106]. Bovolenta et al. [55] reported greater intensity of ewe odor and aroma in low-fat “Pitina”, suggesting that the higher NPN concentration in 40% fat ostrich salami may exert a synergistic effect on gamy flavor perception. At 20 weeks of ripening, a general and significant reduction in gamy odor and flavor was observed, as well as a concomitant reduction in NPN, likely as a consequence of the increase in salt concentration and the further decrease in a_w_, which exert a depressive effect on proteases. Starting from the assumption that the gamy flavor is a complex and multifactorial flavor, it can be speculated that some molecular precursors included in NPN could be lost in the last part of the ripening period for the salami of the present study, with beneficial effects in terms of sensory quality. Metallic flavor was more intense in 40% fat salami and in those samples with lower salt content, with a significant fat × salt interaction at 10 weeks. Ref. [55] instead observed a trend towards higher metallic flavor intensity at lower fat concentration in the fermented meat product “Pitina”. Metallic flavor has been identified as an indicator of a short ripening period in Milano salami [107] and in aged ham [108], but also because of a lower concentration of volatile compounds derived from lipoperoxidation, which may perform a masking function on metallic aroma [109]. In the present study, metallic flavor was significantly correlated with the M:P and WPS in samples with 2.4% salt at 10 weeks of ripening (Appendix A, Table A3 and Table A4), consistent with [110], who reported an increase in metallic flavor intensity in dry-cured sausages with lower salt concentrations. At 20 weeks of ripening, the intensity of metallic flavor perceived in FAT30 and FAT40 ostrich salami was comparable, suggesting that extended ripening attenuated the differential effect of fat content on this attribute.

Contrary to expectations, moldy flavor was more intense in FAT40 salami and in samples with lower salt content at 10 weeks, with significant fat × salt and fat × lab interactions. The higher salt concentration in 30% fat ostrich salami appears to attenuate moldy flavor, possibly through its inhibitory effect on fungal growth and volatile compound production. Bovolenta et al. [55] reported greater intensity of moldy flavor in high-fat “Pitina”, in agreement with the present findings at 10 weeks. At 20 weeks of ripening, moldy odor intensity was significantly higher in 30% fat ostrich salami, consistent with the greater mold coverage observed on the surface of leaner products, as the progressive development of fungal colonies during extended ripening contributed to a more pronounced moldy character in the final product. Mold odor and flavor are associated with 1-octen-3-ol, which exhibits the typical odor of mushroom [111]. These sensory notes have been frequently identified in dry-cured sausages by instrumental analysis [112,113,114,115,116]. Rancid odor and flavor were perceived with low intensity throughout the ripening process, suggesting an overall satisfactory oxidative stability of the product. At 10 weeks of ripening, a significant effect of starter culture type was observed, with LAB8 associated with more intense rancid odor and LAB6 with more intense rancid flavor. It is reasonable to hypothesize that the perception of the rancid flavor was confounded by the simultaneous presence of salty or spicy notes, which may have partially masked or modulated its intensity. The significant correlation between the M:P and WPS and rancid odor in FAT30 ostrich salami with 2.6% salt and LAB8 starter culture at 20 weeks of ripening (Appendix A, Table A3 and Table A4) further supports the role of dehydration and salt concentration in modulating lipid oxidation-derived volatile compounds.

The spicy odor was perceived with greater intensity in 30% fat ostrich salami at 10 weeks of ripening; however, this trend was reversed at 20 weeks, when 40% fat salami received a higher spicy odor score. A similar reversal in the perception of spicy odor as a function of fat content and ripening time has been reported [11,52,59]. The significant correlation between M:P and WPS with spicy odor in FAT30 ostrich salami, combined with the opposing trend observed at 10 and 20 weeks of ripening (Appendix A, Table A3 and Table A4), suggests that the progressive concentration effect associated with dehydration during ripening differentially influences the perception of spice-derived volatile compounds depending on fat content.

Salami with 40% fat, as well as samples with the LAB6 starter culture, exhibited significantly higher off-flavor intensity, with a significant fat × lab interaction effect. The LAB6 group (*Lb. curvatus*) therefore appeared to influence the biosynthesis of compounds that negatively affected the flavor of the ostrich salami. Furthermore, the technological parameters M:P and WPS showed significant positive correlations with off-flavor intensity (Appendix A) in samples containing 2.4% salt, both with the LAB6 starter culture and after 20 weeks of ripening.

Regarding texture, greater tenderness and juiciness were observed in 40% fat salami, with a notable fat × lab interaction effect: juiciness increased significantly in the 30% fat group, whereas it decreased in the 40% fat group for both LAB6 and LAB8 starter cultures. These findings are consistent with previously reported results [11,52,53,55,59], corroborating the well-established relationship between fat content and textural properties in fermented meat products. As highlighted by [12], sensory evaluations of texture vary depending on individual consumer preferences. Regarding ripening length (the persistence of the ripening flavor), it was greater in 30% fat ostrich salami at both ripening time points assessed. Moreover, M:P and WPS significantly influenced ripening length across most of the experimental conditions tested (Appendix A).

### 4.2. Effect of Ripening Period

The effect of the ripening period (10 vs. 20 weeks) on selected sensory descriptors is illustrated in Figure 1 and Figure 2. While rancid odor did not vary significantly between the two assessed time points, rancid flavor intensified at 20 weeks, whereas both odor and the gamy flavor showed a progressive decrease. Conversely, moldy odor increased after 20 weeks of ripening compared to perceptions recorded at 10 weeks. The ripening period effect was similarly detected for salty taste, fatty flavor, and the flavor persistency, whose perceived intensity increased at 20 weeks of ripening. In contrast, overall odor intensity decreased as a function of the ripening period. Color homogeneity and fat–lean cohesion improved progressively with ripening length, while tenderness and juiciness showed a declining trend. With respect to the physicochemical indicators, the mean variations observed between 10 and 20 weeks of ripening are reported in Table 7. A significant increase in pH was observed, consistent with the behavior typically reported for non-acidic salami [117], alongside an increase in FFA, as documented in previous studies [11,52,53,67,118,119,120]. As expected, both salt percentage and the WPS, increased because of weight loss occurring throughout the processing phase [39]. TBARs values did not exhibit significant variations, contrary to observations reported by other authors [52,53,57,67], in whose studies the secondary oxidation indicator increased progressively from the onset to the conclusion of the ripening process. Notably, in a study on foal dry-cured sausage produced with varying levels of pork fat, it was reported that TBARs values increased up to day 28, after which they stabilized or declined during the subsequent 3 weeks of processing [11].

### 4.3. Principal Component Analysis

**Figure A1:**
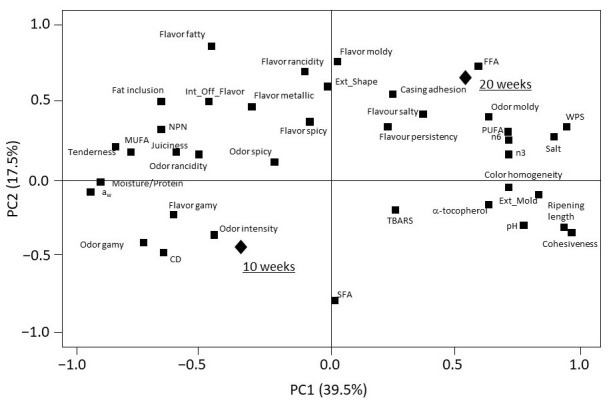
Relationships between ostrich salami manufactured with two different levels of pork back fat, sodium chloride and starter cultures and the studied physicochemical, fatty acid and sensory parameters obtained by PCA. Projection of the variables in the plane defined by the first two principal components.

To investigate correlations among the variables under study, principal component analysis (PCA) was performed, with results summarized in Appendix A, Figure A1. The PCA revealed that 57% of the total variability was accounted for by the first two principal components. The Kaiser–Meyer–Olkin (KMO) measure and Bartlett’s test of sphericity yielded values of 0.73 and *p* < 0.001, respectively, while factor loadings for each variable are shown in Appendix A, Table A5. Principal component 1 (PC1) explained 39.5% of the total variability and showed positive associations with salt, pH, PUFA, *n*-6, *n*-3 FA, and WPS and negative associations with a_w_, the M:P, and the MUFA summation among the physicochemical parameters. Among the sensory variables, PC1 was positively associated with fat–lean cohesion, flavor persistency, and external mold and negatively associated with tenderness, fat inclusion, and odor intensity. Principal component 2 (PC2) accounted for 17.5% of the total variability, showing positive correlations with flavor fatness among sensory variables and negative correlations with the sum of SFA among the chemical variables. PC1 effectively discriminated samples with 40% fat inclusion (negative PC1, positive PC2 quadrant) from those with 30% fat inclusion (positive PC1, negative PC2 quadrant), while PC2 separated samples ripened for 20 weeks (positive PC1 and PC2 quadrant) from those ripened for 10 weeks (negative PC1 and PC2 quadrant). Notably, neither salt percentage nor starter culture type contributed substantially to explaining the observed variability. Salami samples with 30% fat inclusion were characterized by higher pH values, greater color homogeneity, improved fat–lean cohesion, and enhanced flavor persistency, whereas salami samples with 40% fat inclusion exhibited greater intensity of off-flavors, rancid flavor, and fatty flavor, along with higher tenderness, elevated MUFA, and increased proteolysis (NPN). At 10 weeks of ripening, ostrich salami displayed a higher M:P ratio and a_w_, more intense odor, and greater gamy odor and flavor, whereas after 20 weeks of ripening, ostrich salami samples were characterized by higher concentrations of PUFA (*n*-6 and *n*-3), FFA, salt, and WPS, as well as a greater intensity of moldy odor and flavor and salty taste.

The M:P and WPS showed significant variations between the two ripening periods. Among the factors considered in the present study PCA clearly discriminated salami by percentage of fat inclusion, for which the chemical variables of M:P, WPS, and a_w_ emerged as the most effective and predictive. This is consistent with the marked predisposition of the muscle fraction to lose water during ripening, with a consequent increase in salt concentration and decrease in moisture, in agreement with findings reported by Casiraghi et al. [121]. On the other hand according to the length of ripening, FFA and moldy and fatty flavor were predictive of 20 weeks of ripening, whereas SFA was related to salami at 10 weeks of ripening.

## 5. Conclusions and Future Implications

This study evaluated the effects of two levels of pork back-fat inclusion (30% and 40%), two sodium chloride concentrations (2.4% and 2.6%), and two starter culture combinations (*Lactobacillus curvatus* + *Staphylococcus xylosus* and Lactobacillus sakei + *Staphylococcus xylosus*) on the physicochemical and sensory characteristics of ostrich-meat salami assessed after 10 and 20 weeks of ripening. Overall, results of the present research should be interpreted in the context of a manufacturing trial because the present experiment was structured with a single-batch-per-treatment-combination (due to structural-processing limitations in the production facility, which was an artisanal laboratory and not industry plant). Therefore, extrapolation to a wider population of production batches should be made with caution and possibly validated with an industrial-type experiment.

The formulation containing 30% pork back fat and 2.4% sodium chloride produced a stable product, as evidenced by satisfactory pH and water activity (a_w_) values after only 10 weeks of ripening, together with moderate levels of free fatty acids (FFA) and non-protein nitrogen (NPN). This formulation also promoted surface fungal development, good color homogeneity, and enhanced product cohesiveness. Furthermore, it was characterized by very low intensities of undesirable odors and flavors, a more pronounced perception of ripening and maturity, and desirable textural attributes, including adequate juiciness and tenderness. The starter culture containing *Lb. sakei* exhibited greater proteolytic activity than the culture containing *Lb. curvatus*. Among the three experimental factors investigated, pork back-fat inclusion level had the greatest influence on both physicochemical and sensory characteristics. The main limitation of this study is the absence of microbiological analyses. Such analyses would have been useful not only for monitoring the growth dynamics of the starter cultures, but also for identifying indigenous microbial populations associated with the lactic acid microbiota that may develop during the post-fermentation ripening stage and contribute to the sensory characteristics of the salami. Furthermore, the results suggest that formulating the mixture with less than 30% pork back fat is not advisable, as it may excessively accentuate certain characteristic sensory attributes of ostrich meat, including an overly intense color and excessively pronounced spicy, salty, and species-specific odors and flavors. It is well-established that nitrate and nitrite salts, in addition to their antimicrobial properties, act as effective antioxidants in processed meat products. The present study demonstrated that Italian-style salami can be successfully produced from ostrich meat without the addition of nitrate or nitrite salts, yielding a product with acceptable physicochemical and sensory characteristics. Product quality was influenced by the level of pork back-fat inclusion, sodium chloride concentration, and starter culture employed.

Based on these findings, future studies should replicate the formulation that shows the most favorable physicochemical and sensory performance and conduct challenge tests against spore-forming microorganisms, particularly *Clostridium* species. Additional investigations should include the characterization of volatile compounds to explore potential relationships between lipid oxidation products, microbial fermentation metabolites, and protein hydrolysis products, and their contribution to the sensory profile of the salami. Finally, consumer acceptance studies should be performed to assess the market potential and overall acceptability of the product.

## Figures and Tables

**Table 1:** Effects of two levels of fat (30 vs. 40%), salt (2.4 vs. 2.6%) and LAB starter cultures (LAB6: *Lb. curvatus*/*S. xylosus* vs. LAB8: *Lb. sakei*/*S. xylosus*) and their interaction on chemical parameters of ostrich salami, measured at 10 weeks of ripening.

Items	FAT (F)		SALT (S)		LAB (L)		*p*-Values							RSD ^1^
	30	40	2.4	2.6	6	8	F	S	L	F × S	F × L	S × L	F × S × L	
N. salami	4	4	4	4	4	4								
pH	5.48	5.43	5.50	5.41	5.46	5.46	0.013	<0.001	ns	0.005	ns	0.009	ns	0.05
a_w_	0.86	0.89	0.88	0.87	0.87	0.88	<0.0001	<0.0001	<0.0001	0.0327	0.0230	ns	<0.0001	0.002
NaCl (g/100 g sample)	3.73	3.21	3.25	3.69	3.39	3.56	<0.0001	<0.0001	0.0069	0.0165	ns	ns	0.0165	0.16
M:P ^2^	1.29	1.75	1.54	1.50	1.49	1.55	<0.0001	0.0203	0.0009	<0.0001	<0.0001	ns	0.0015	0.04
WPS (%) ^3^	10.1	8.1	8.6	9.6	9.1	9.1	<0.001	0.011	ns	ns	ns	ns	ns	0.75
alpha-tocopherol (mg/kg)	0.40	0.30	0.35	0.34	0.35	0.35	<0.0001	ns	ns	ns	ns	ns	ns	0.04
FFA (g oleic acid/100 g fat) ^4^	9.09	7.51	8.62	7.97	8.47	8.12	<0.0001	0.0007	0.0447	0.0433	ns	0.0063	0.0004	0.47
CD (Absorbance unit) ^5^	0.006	0.008	0.009	0.005	0.006	0.008	<0.0001	<0.0001	<0.0001	<0.0001	<0.0001	<0.0001	<0.0001	0.0003
TBARs (mg MDA/kg) ^6^	0.36	0.21	0.28	0.29	0.28	0.29	<0.0001	ns	ns	ns	ns	<0.0001	<0.0001	0.05
NPN (soluble N/P, %) ^7^	15.1	18.4	16.8	16.7	16.2	17.3	<0.0001	ns	<0.0001	ns	<0.0001	<0.0001	<0.0001	0.32

^1^ Residual standard deviation; ^2^ Moisture:Protein ratio; ^3^ Water Phase Salt; ^4^ Free Fatty Acids; ^5^ Conjugated diene; ^6^ Thiobarbituric Acid Reactive substances; ^7^ Non-Protein Nitrogen.

**Table 2:** Effects of two levels of fat (30 vs. 40%), salt (2.4 vs. 2.6%) and LAB starter cultures (LAB6: *Lb. curvatus*/*S. xylosus* vs. LAB8: *Lb. sakei*/*S. xylosus*) and their interaction on chemical parameters of ostrich salami, measured at 20 weeks of ripening.

Items	FAT (F)		SALT (S)		LAB (L)		*p*-Values				RSD ^1^
	30	40	2.4	2.6	6	8	F	S	L	F × L	
N. salami	5	5	5	5	5	5					
pH	5.61	5.38	5.51	5.49	5.47	5.52	<0.0001	ns	0.0021	0.0271	0.04
a_w_	0.80	0.87	0.84	0.82	0.83	0.83	<0.0001	0.0074	ns	ns	0.02
NaCl (g/100 g sample)	4.56	4.00	4.12	4.44	4.28	4.29	<0.0001	0.0106	ns	ns	0.33
M:P ^2^	0.96	1.45	1.22	1.19	1.19	1.23	<0.0001	ns	ns	ns	0.13
WPS (%) ^3^	14.8	11.5	12.6	13.6	13.4	12.9	<0.001	ns	ns	ns	1.4
alpha-tocopherol (mg/kg)	0.42	0.35	0.40	0.37	0.39	0.38	0.0199	ns	ns	ns	0.08
FFA (g oleic acid/100 g fat) ^4^	13.0	12.9	13.1	12.7	13.2	12.6	ns	ns	ns	ns	1.6
CD (Absorbance unit) ^5^	<LOQ	<LOQ	<LOQ	<LOQ	<LOQ	<LOQ	-	-	-	-	-
TBARs (mg MDA/kg) ^6^	0.34	0.22	0.27	0.29	0.27	0.29	0.0108	ns	ns	Ns	0.12
NPN (soluble N/P, %) ^7^	14.9	16.9	16.1	15.7	16.3	15.5	0.0029	ns	ns	ns	1.70
Heme iron (mg/kg) ^8^	12.4	8.80	10.4	10.9	10.8	10.5	<0.0001	ns	ns	ns	1.09

^1^ Residual standard deviation; ^2^ Moisture:Protein ratio; ^3^ Water Phase Salt; ^4^ Free Fatty Acids; ^5^ Conjugated diene; ^6^ Thiobarbituric Acid Reactive substances; ^7^ Non-Protein Nitrogen; ^8^ Heme iron in the salami batter: 14.9 mg/kg meat.

**Table 3:** Effects of two levels of fat (30 vs. 40%), salt (2.4 vs. 2.6%) and LAB starter cultures (LAB6: *Lb. curvatus*/*S. xylosus* vs. LAB8: *Lb. sakei*/*S. xylosus*) and their interaction on fatty acid content (% of the total FAME) of ostrich salami ripened for 10 weeks.

Items	FAT (F)		SALT (S)		LAB (L)		*p*-Values					RSD ^1^
	30	40	2.4	2.6	6	8	F	S	L	F × S	F × L	
N. salami	4	4	4	4	4	4						
C14:0	1.36	1.34	1.35	1.35	1.38	1.33	0.0471	ns	<0.0001	ns	0.0219	0.03
C16:0	24.4	23.9	24.0	24.2	24.4	23.90	<0.0001	ns	0.0012	ns	ns	0.33
C18:0	13.3	12.9	13.1	13.1	13.1	13.10	<0.0001	ns	ns	ns	0.0238	0.16
Others	0.75	0.70	0.73	0.72	0.72	0.73	0.0021	ns	ns	ns	ns	0.04
Σ SFA	39.8	38.8	39.2	39.5	39.5	39.1	<0.0001	ns	0.0080	ns	ns	0.44
C16:1	2.12	2.04	2.07	2.09	2.09	2.07	<0.0001	ns	ns	0.0318	0.0394	0.04
C18:1 *n*-9	39.8	41.1	40.5	40.5	40.5	40.5	<0.0001	ns	ns	ns	ns	0.32
C18:1 *n*-11	2.92	2.97	2.96	2.93	2.94	2.95	ns	ns	ns	ns	ns	0.14
Σ Others	0.99	1.00	0.99	1.00	0.99	1.00	ns	ns	ns	ns	ns	0.05
Σ MUFA	45.8	47.2	46.5	46.5	46.5	46.5	<0.0001	ns	ns	ns	ns	0.41
C18:2 *n*-6	10.4	10.0	10.2	10.2	10.2	10.2	0.0066	ns	ns	ns	ns	0.41
C18:3 *n*-6	0.03	0.03	0.03	0.02	0.03	0.03	ns	ns	ns	ns	ns	0.01
C20:2 *n*-6	0.50	0.51	0.51	0.51	0.50	0.51	ns	ns	ns	ns	ns	0.04
C20:4 *n*-6	0.12	0.11	0.12	0.12	0.11	0.12	ns	ns	ns	ns	ns	0.04
C18:3 *n*-3	0.50	0.45	0.46	0.48	0.47	0.48	0.0003	ns	ns	0.0151	ns	0.04
Σ Others	0.13	0.13	0.14	0.12	0.13	0.13	ns	ns	ns	ns	ns	0.04
Σ PUFA	11.7	11.2	11.4	11.5	11.4	11.5	0.0199	ns	ns	ns	ns	0.53
SFA:UFA	0.69	0.66	0.68	0.68	0.68	0.67	<0.0001	ns	0.0144	ns	ns	0.01
MUFA:PUFA	3.92	4.21	4.07	4.06	4.08	4.05	0.0005	ns	ns	ns	ns	0.20
PUFA:SFA	0.29	0.29	0.29	0.29	0.29	0.29	ns	ns	ns	ns	ns	0.01
Σ *n*-6	10.6	10.2	10.4	10.4	10.4	10.4	0.0151	ns	ns	ns	ns	0.47
Σ *n*-3	0.56	0.51	0.53	0.54	0.53	0.54	0.0038	ns	ns	0.0464	ns	0.05
*n*-6:*n*-3	19.0	20.1	19.8	19.3	19.7	19.4	0.0488	ns	ns	0.0177	ns	1.50
AI	0.53	0.51	0.51	0.52	0.52	0.51	<0.0001	ns	0.0007	ns	ns	0.01
TI	1.30	1.26	1.28	1.29	1.29	1.27	<0.0001	ns	0.0152	ns	ns	0.02
hH	1.97	2.05	2.02	2.01	1.99	2.03	<0.0001	ns	0.0037	ns	ns	0.04

^1^ Residual standard deviation; SFA: Saturated Fatty Acids; MUFA: Monounsaturated Fatty Acids; PUFA: Polyunsaturated Fatty Acids; AI = (C12:0 + 4×C14:0 + C16:0)/[(∑MUFA + ∑PUFA(*n*-6) + (*n*-3))]; TI = (C14:0 + C16:0 + C18:0)/[0.5 × ∑MUFA + 0.5 × ∑PUFA (*n*-6) + 3 × ∑PUFA (*n*-3) + (*n*-3)/(*n*-6)]; hH = [C18:1(*n*-9) + 18:2(*n*-6) + 18:3(*n*-6)/(C12:0 + C14:0 + C16:0) + (C18:3(*n*-3) + C18:4(*n*-3) + C20:4(*n*-6)/(C12:0 + C14:0 + C16:0)] [51].

**Table 4:** Effects of two levels of fat (30 vs. 40%), salt (2.4 vs. 2.6%) and LAB starter cultures (LAB6: *Lb. curvatus*/*S. xylosus* vs. LAB8: *Lb. sakei*/*S. xylosus*) and their interaction on fatty acids content (% of the total FAME) of ostrich salami ripened for 20 weeks.

Items	FAT (F)		SALT (S)		LAB (L)		*p*-Values					RSD ^1^
	30	40	2.4	2.6	6	8	F	S	L	F × S	F × L	
N. salami	5	5	5	5	5	5						
C14:0	1.36	1.40	1.38	1.38	1.37	1.39	0.0078	ns	ns	ns	ns	0.04
C16:0	23.5	23.3	23.4	23.4	23.4	23.5	0.0413	ns	ns	ns	ns	0.26
C18:0	13.2	12.8	13.1	13.0	13.1	13.0	<0.0001	0.0478	ns	0.0313	ns	0.18
Σ Others	0.76	0.71	0.73	0.73	0.74	0.73	0.0057	ns	ns	ns	ns	0.04
Σ SFA	38.8	38.2	38.6	38.5	38.5	38.5	0.0003	ns	ns	ns	ns	0.37
C16:1	2.31	2.25	2.25	2.30	2.26	2.29	0.0002	0.0027	ns	ns	ns	0.04
C18:1 *n*-9	39.3	40.4	39.8	39.9	39.9	39.8	<0.0001	ns	ns	ns	ns	0.25
C18:1 *n*-11	2.67	2.62	2.65	2.64	2.66	2.63	ns	ns	ns	ns	ns	0.12
Σ Others	1.28	1.27	1.26	1.30	1.28	1.27	ns	ns	ns	ns	ns	0.07
Σ MUFA	45.6	46.5	45.9	46.2	46.1	46.0	<0.0001	0.0088	ns	ns	0.0343	0.23
C18:2 *n*-6	10.9	10.6	10.7	10.8	10.6	10.80	ns	ns	ns	ns	ns	0.39
C20:2 *n*-6	0.52	0.53	0.53	0.53	0.52	0.53	ns	ns	ns	ns	ns	0.02
C18:3 *n*-6	0.02	0.02	0.02	0.02	0.02	0.02	ns	ns	ns	ns	ns	0.00
C20:4 *n*-6	0.23	0.21	0.22	0.22	0.21	0.23	ns	ns	ns	ns	0.0482	0.04
C18:3 *n*-3	0.55	0.51	0.53	0.53	0.52	0.54	0.0019	ns	ns	ns	ns	0.03
Σ Others	0.18	0.17	0.17	0.18	0.18	0.18	ns	ns	ns	ns	ns	0.03
Σ PUFA	12.3	12.0	12.2	12.2	12.1	12.3	ns	ns	ns	ns	ns	0.48
SFA:UFA	0.67	0.65	0.66	0.66	0.66	0.66	<0.001	ns	ns	ns	ns	0.03
MUFA:PUFA	3.70	3.87	3.79	3.78	3.82	3.75	0.0053	ns	ns	ns	ns	0.15
PUFA:SFA	0.32	0.31	0.32	0.32	0.31	0.32	ns	ns	ns	ns	ns	0.02
Σ *n*-6	11.2	10.9	11.0	11.1	10.9	11.1	ns	ns	ns	ns	ns	0.42
Σ *n*-3	0.62	0.58	0.60	0.60	0.59	0.60	0.0085	ns	ns	ns	ns	0.04
*n*-6:*n*-3	18.1	18.9	18.5	18.5	18.4	18.5	0.0056	ns	ns	ns	ns	0.77
AI	0.51	0.50	0.50	0.50	0.50	0.50	ns	ns	ns	ns	0.0204	0.01
TI	1.26	1.23	1.25	1.24	1.25	1.24	0.0062	ns	ns	ns	ns	0.02
hH	2.05	2.09	2.07	2.08	2.08	2.07	0.0042	ns	ns	ns	0.0467	0.04

^1^ Residual standard deviation; SFA: Saturated Fatty Acids; MUFA: Monounsaturated Fatty Acids; PUFA: Polyunsaturated Fatty Acids; AI = (C12:0 + 4×C14:0 + C16:0)/[(∑MUFA + ∑PUFA(*n*-6) + (*n*-3))]; TI = (C14:0 + C16:0 + C18:0)/[0.5 × ∑MUFA + 0.5 × ∑PUFA (*n*-6) + 3 × ∑PUFA (*n*-3) + (*n*-3)/(*n*-6)]; hH = [C18:1(*n*-9) + 18:2(*n*-6) + 18:3(*n*-6)/(C12:0 + C14:0 + C16:0) + (C18:3(*n*-3) + C18:4(*n*-3) + C20:4(*n*-6)/(C12:0 + C14:0 + C16:0)] [51].

**Table 5:** Effects of two levels of fat (30 vs. 40%), salt (2.4 vs. 2.6%) and LAB starter cultures (LAB6: *Lb. curvatus*/*S. xylosus* vs. LAB8: *Lb. sakei*/*S. xylosus*) and their interaction on sensory attributes of ostrich salami ripened for 10 weeks.

Items	FAT (F)		SALT (S)		LAB (L)		*p*-Values						RSD ^1^
	30	40	2.4	2.6	6	8	F	S	L	F × S	F × L	S × L	
**External appearance:**													
Shape regularity	11.2	12.8	12.17	11.8	11.3	12.7	0.001	ns	0.006	ns	<0.001	ns	1.34
Casing adhesion	11.2	12.3	12.5	11.0	10.8	12.7	<0.05	0.001	<0.001	ns	0.01	ns	1.34
Mold growth	1.95	0.78	1.33	1.40	1.06	1.67	<0.001	ns	ns	ns	<0.001	ns	0.76
**Internal appearance:**													
Color homogeneity	11.0	10.7	9.98	11.7	10.6	11.07	ns	<0.001	ns	<0.001	ns	0.05	1.2
Cohesiveness	10.1	5.73	7.01	8.85	7.74	8.11	<0.001	ns	ns	0.004	ns	ns	1.6
Fat inclusion	10.3	11.4	11.4	10.3	10.8	11.0	<0.001	0.001	ns	0.009	ns	0.001	0.78
**Odor:**													
Intensity	11.1	11.3	10.7	11.7	11.0	11.4	ns	<0.001	0.05	ns	ns	<0.001	0.7
Moldy	0.38	0.37	0.34	0.41	0.32	0.43	ns	ns	0.037	ns	<0.001	0.001	0.15
Rancidity	1.54	1.81	1.30	2.05	1.48	1.87	ns	<0.001	0.047	ns	ns	ns	0.55
Gamy	2.64	3.42	3.00	3.06	2.85	3.21	<0.001	ns	ns	0.007	0.003	ns	0.39
Spicy	9.14	8.16	8.62	8.69	8.73	8.58	0.005	ns	ns	ns	ns	<0.001	0.91
**Flavor:**													
Gamy	2.47	3.38	3.03	2.82	3.02	2.82	<0.001	ns	ns	0.003	<0.001	ns	0.44
Salty	9.56	8.50	8.83	9.23	9.14	8.92	<0.001	ns	ns	0.034	ns	0.01	0.42
Rancidity	1.41	1.35	1.36	1.39	1.58	1.18	ns	ns	0.002	ns	0.005	ns	0.39
Metallic	3.16	3.68	3.72	3.12	3.35	3.49	0.006	0.001	ns	0.004	ns	ns	0.5
Fatness	6.05	8.39	7.72	6.72	7.46	6.98	<0.001	0.048	ns	ns	ns	0.017	0.83
Moldy	0.47	1.63	1.64	0.46	1.31	0.79	0.001	0.001	ns	<0.001	0.002	ns	0.93
Spicy	8.35	7.86	8.12	8.09	8.25	7.95	ns	ns	ns	ns	ns	<0.001	0.8
Flavor persistency	10.2	9.69	9.96	9.90	10.2	9.63	ns	ns	0.019	<0.001	ns	ns	0.72
**Off flavors:**													
Intensity	4.02	5.21	4.62	4.61	5.24	3.99	<0.001	ns	<0.001	ns	<0.001	ns	0.8
**Texture:**													
Tenderness	8.78	12.6	10.7	10.7	10.2	11.2	<0.001	ns	ns	0.008	ns	ns	0.66
Juiciness	9.39	11.3	10.4	10.3	10.4	10.3	<0.001	ns	ns	ns	<0.001	ns	0.58
**Perception of the degree of maturity**	5.21	3.83	4.31	4.74	4.43	4.61	<0.001	ns	ns	ns	0.003	ns	0.37

^1^ Residual standard deviation.

**Table 6:** Effects of two levels of fat (30 vs. 40%), salt (2.4 vs. 2.6%) and LAB starter cultures (LAB6: *Lb. curvatus*/*S. xylosus* vs. LAB8: *Lb. sakei*/*S. xylosus*) and their interaction on sensory attributes of ostrich salami ripened for 20 weeks.

Items	FAT (F)		SALT (S)		LAB (L)		*p*-Values				RSD ^1^
	30	40	2.4	2.6	6	8	F	S	L	F × S	
**External appearance:**											
Shape regularity	12.7	12.7	12.9	12.6	12.9	12.58	ns	ns	ns	ns	0.57
Casing adhesion	13.3	13.4	13.5	13.2	13.3	13.4	ns	ns	ns	ns	0.45
Mold growth	3.53	1.08	2.30	2.31	2.28	2.33	<0.001	ns	ns	ns	0.75
**Internal appearance:**											
Color homogeneity	12.8	11.3	11.8	12.3	12.1	12.0	<0.001	ns	ns	ns	0.86
Cohesiveness	12.8	6.89	9.23	10.5	10.1	9.65	<0.001	ns	ns	0.004	1.88
Fat inclusion	9.49	11.5	10.7	10.3	10.5	10.5	<0.001	ns	ns	ns	0.94
**Odor:**											
Intensity	10.2	10.4	10.4	10.2	10.3	10.3	ns	ns	ns	ns	0.94
Moldy	1.76	1.06	1.35	1.47	1.45	1.37	<0.001	ns	ns	ns	0.48
Rancidity	1.10	1.96	1.37	1.68	1.46	1.59	0.007	ns	ns	ns	0.84
Gamy	1.89	2.01	1.98	1.92	2.03	1.87	ns	ns	ns	ns	0.58
Spicy	8.35	9.18	8.88	8.65	8.66	8.87	0.001	ns	ns	ns	0.66
**Flavor:**											
Gamy	2.23	2.44	2.33	2.34	2.31	2.36	ns	ns	ns	ns	0.59
Salty	10.3	10.1	10.0	10.4	10.4	10.1	ns	ns	ns	ns	0.78
Rancidity	1.37	2.44	1.93	1.88	2.01	1.81	0.003	ns	ns	ns	0.95
Metallic	3.54	3.68	3.56	3.66	3.71	3.51	ns	ns	ns	ns	0.57
Fatness	7.07	10.1	8.94	8.23	8.59	8.57	<0.001	ns	ns	ns	0.79
Moldy	2.35	2.95	2.84	2.46	2.81	2.49	ns	ns	ns	ns	1.22
Spicy	8.13	8.73	8.27	8.59	8.24	8.63	0.026	ns	ns	ns	0.73
Flavor persistency	10.2	10.3	10.2	10.4	10.3	10.3	ns	ns	ns	ns	0.59
**Off flavors:**											
Intensity	4.10	5.36	4.53	4.92	4.72	4.73	0.002	ns	ns	ns	1.08
**Texture:**											
Tenderness	6.68	11.47	8.82	9.33	9.09	9.06	<0.001	ns	ns	ns	0.92
Juiciness	9.52	10.4	9.86	10.1	9.94	10.0	<0.001	ns	ns	ns	0.67
**Perception of the degree of maturity**	7.45	3.52	5.11	5.87	5.54	5.44	<0.001	ns	ns	0.005	0.82

^1^ Residual standard deviation.

**Table 7:** Variation of physicochemical, lipid oxidation, proteolysis and fatty acid indicators between 10 and 20 weeks of ripening in selected traits.

Items	*Indices that increased as a function of the ripening period*									
Weeks	pH	FFA ^1^	TBARs ^2^	Salt ^3^	alpha-tocopherol ^4^	WPS ^5^	Σ PUFA ^6^	Σ *n*-6 ^7^	Σ *n*-3 ^8^	PUFA/SFA
10	5.48	8.20	0.27	3.51	0.36	9.21	11.5	10.5	0.54	0.29
20	5.52	12.9	0.27	4.33	0.40	13.4	12.2	11.0	0.60	0.32
*p*-value	ns	<0.001	ns	<0.001	ns	<0.001	<0.001	<0.001	<0.001	<0.001
RSD ^13^	0.10	1.32	0.16	0.42	0.08	1.78	0.51	0.45	0.05	0.01
	*Indices that decreased as a function of the ripening period*									
Weeks		NPN ^9^	NPN ^10^	a_w_	Moisture:Protein	Σ SFA ^11^	Σ MUFA ^12^	MUFA/PUFA	SFA/UFA	
10		16.5	0.94	0.87	1.50	39.4	46.4	4.04	0.68	
20		15.6	0.86	0.83	1.17	38.5	46.0	3.77	0.66	
*p*-value		ns	<0.001	<0.001	<0.001	<0.001	0.011	<0.001	<0.001	
RSD ^13^		2.05	0.04	0.03	0.26	0.60	0.67	0.20	0.02	

^1^ g oleic acid/100 g fat; ^2^ mg MDA/kg; ^3^ g/100 g; ^4^ mg/kg; ^5^ g/100 g; ^6^ % total FAME; ^7^ % total FAME; ^8^ % total FAME; ^9^ Soluble N 12% TCA/P % fresh weight; ^10^ Soluble N 12% TCA/P % dry matter: ^11^ % total FAME; ^12^ % total FAME; ^13^ Residual Standard Deviation; ns (*p* > 0.05).

**Figure A2:**
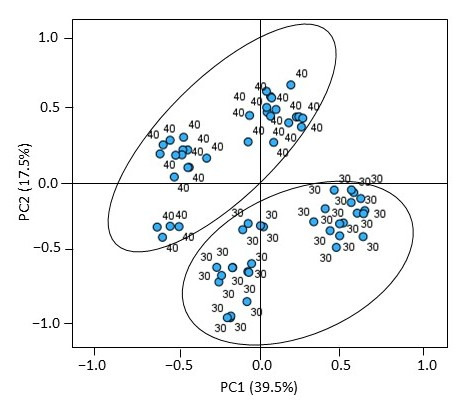
Projection of the samples in the plane defined by the first two principal components according to the percentage of fat inclusion (30% vs. 40%)**.**

**Figure A3:**
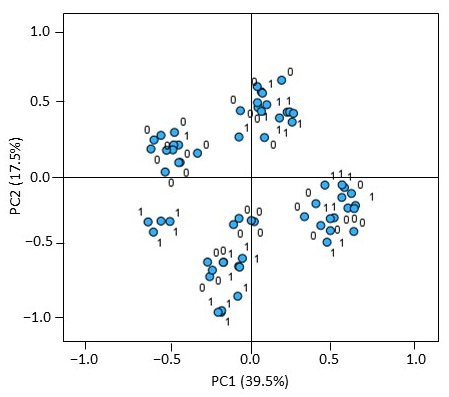
Projection of the samples in the plane defined by the first two principal components according to the percentage of salt inclusion (0 = 2.4% vs. 1 = 2.6%).

**Figure A4:**
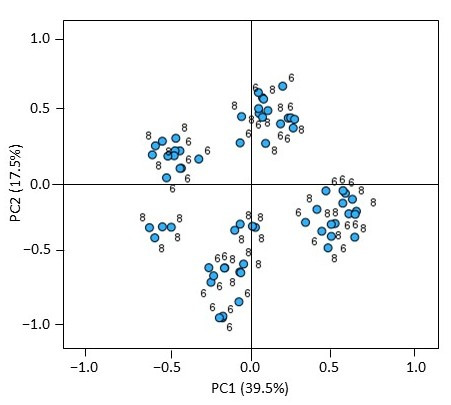
Projection of the samples in the plane defined by the first two principal components according to the starter culture (6 = LAB6, 8 = LAB8).

## Data Availability

The raw data supporting the conclusions of this article will be made available by the authors on request.

## Appendix A

**Table A1:** Effect of two levels of fat, salt and LAB starter cultures and their interactions on fatty acid content (g/100 g salami) of ostrich salami ripened for 10 weeks.

Items	FAT (F)		SALT (S)		LAB (L)		*p*-Values					RSD ^1^
	30	40	2.4	2.6	6	8	F	S	L	F × S	F × L	
N. salami	4	4	4	4	4	4						
C14:0	0.44	0.47	0.46	0.45	0.47	0.43	<0.0001	ns	<0.0001	0.008	0.0479	0.02
C16:0	7.84	8.34	8.12	8.06	8.40	7.78	<0.0001	ns	<0.0001	0.008	0.0008	0.28
C18:0	4.26	4.51	4.41	4.36	4.52	4.26	0.0003	ns	0.0002	0.0285	0.0003	0.17
Σ Others	0.24	0.24	0.25	0.24	0.25	0.24	ns	ns	0.0386	ns	ns	0.02
Σ SFA	12.8	13.6	13.2	13.1	13.6	12.7	<0.0001	ns	<0.0001	0.0122	0.0006	0.46
C16:1	0.68	0.71	0.70	0.70	0.72	0.67	0.0037	ns	0.0001	0.0052	0.0367	0.03
C18:1 *n*-9	12.8	14.4	13.7	13.5	14	13.2	<0.0001	ns	0.0002	0.0128	0.0012	0.52
C18:1 *n*-11	0.94	1.04	1.00	0.97	1.02	0.96	<0.0001	ns	0.0005	0.0067	0.0013	0.04
Σ Others	0.22	0.26	0.24	0.24	0.25	0.23	<0.0001	ns	ns	ns	ns	0.02
Σ MUFA	14.7	16.5	15.7	15.5	16.1	15.1	<0.0001	ns	0.0001	0.0092	0.001	0.58
C18:2 *n*-6	3.35	3.50	3.45	3.40	3.52	3.33	ns	ns	0.0424	ns	ns	0.25
C20:2 *n*-6	0.16	0.18	0.17	0.17	0.17	0.17	0.0121	ns	ns	ns	ns	0.02
C18:3 *n*-6	0.01	0.01	0.01	0.01	0.01	0.01	ns	ns	ns	ns	ns	0.00
C20:4 *n*-6	0.04	0.04	0.04	0.04	0.04	0.04	ns	ns	ns	ns	ns	0.02
C18:3 *n*-3	0.16	0.16	0.16	0.16	0.16	0.16	ns	ns	ns	0.0044	ns	0.01
Σ PUFA	3.76	3.93	3.87	3.82	3.94	3.74	ns	ns	ns	ns	ns	0.31
Σ Others	0.04	0.05	0.05	0.04	0.05	0.04	ns	ns	ns	ns	ns	0.01
Σ *n*-6	3.41	3.56	3.51	3.46	3.58	3.39	ns	ns	ns	ns	ns	0.27
Σ *n*-3	0.18	0.18	0.18	0.18	0.18	0.18	ns	ns	ns	0.0116	ns	0.02

^1^ Residual standard deviation.

**Table A2:** Effect of two levels of fat, salt and LAB starter cultures and their interactions on fatty acid content (g/100 g salami) of ostrich salami ripened for 20 weeks.

Items	FAT		SALT		LAB		*p*-Values			RSD ^1^
	30	40	2.4	2.6	6	8	F	S	L	
N. salami	5	5	5	5	5	5				
C14:0	0.49	0.50	0.50	0.50	0.50	0.49	ns	ns	ns	0.05
C16:0	8.40	8.40	8.40	8.38	8.49	8.29	ns	ns	ns	0.82
C18:0	4.72	4.61	4.70	4.63	4.75	4.59	ns	ns	ns	0.46
Σ Others	0.27	0.27	0.26	0.26	0.27	0.26	ns	ns	ns	0.03
Σ SFA	13.9	13.8	13.9	13.7	14.0	13.6	ns	ns	ns	1.36
C16:1	0.83	0.81	0.81	0.83	0.82	0.81	ns	ns	ns	0.08
C18:1 *n*-9	14.1	14.5	14.3	14.3	14.5	14.1	ns	ns	ns	1.35
C18:1 *n*-11	0.95	0.94	0.95	0.94	0.97	0.93	ns	ns	ns	0.10
Σ Others	0.35	0.36	0.35	0.37	0.37	0.35	ns	ns	ns	0.04
Σ MUFA	16.3	16.7	16.5	16.5	16.8	16.3	ns	ns	ns	1.56
C18:2 *n*-6	3.88	3.81	3.84	3.85	3.87	3.82	ns	ns	ns	0.41
C20:2 *n*-6	0.19	0.19	0.19	0.19	0.19	0.19	ns	ns	ns	0.02
C18:3 *n*-6	0.01	0.01	0.01	0.01	0.01	0.01	ns	ns	ns	0.00
C20:4 *n*-6	0.08	0.08	0.08	0.08	0.08	0.08	ns	ns	ns	0.02
C18:3 *n*-3	0.20	0.18	0.19	0.19	0.19	0.19	ns	ns	ns	0.02
Σ Others	0.06	0.06	0.06	0.06	0.06	0.06	ns	ns	ns	0.01
Σ PUFA	4.41	4.33	4.37	4.38	4.40	4.35	ns	ns	ns	0.48
Σ *n*-6	3.99	3.92	3.95	3.96	3.97	3.93	ns	ns	ns	0.43
Σ *n*-3	0.22	0.21	0.22	0.21	0.22	0.21	ns	ns	ns	0.03

^1^ Residual standard deviation.

**Table A3:** Pearson’s correlation coefficients between moisture:protein (M:P) and sensory scores for the salami, calculated within storage times (fat inclusion, salt and starter cultures) and within treatments (10 and 20 weeks).

Sensory Attributes	Fat Inclusion (%)		Salt (%)		Starter Culture		Weeks	
	30	40	2.4	2.6	6	8	10	20
Color homogeneity	−0.444 **	−0.465 **	−0.487 **	−0.750 **	−0.542 **	−0.578 **	−0.064	−0.746
Cohesiveness	−0.545 **	−0.414 *	−0.832 **	−0.797 **	−0.738 **	−0.860 **	−0.711 **	−0.858 **
Odor intensity	0.542 **	0.284	0.073	0.647 **	0.037	0.662 **	0.059	0.111
Odor rancidity	0.471 **	−0.045	0.406 *	0.580 **	0.304	0.473 **	0.099	0.560 **
Odor gamy	0.808 **	0.732 **	0.643 **	0.760 **	0.605 **	0.726 **	0.792 **	0.268
Odor spicy	0.691 **	−0.445 *	−0.147	0.195	−0.060	−0.102	−0.592 **	0.589 **
Flavor metallic	−0.267	0.090	0.663 **	−0.194	0.079	0.295	0.483 **	0.205
Intensity off-flavor	0.087	−0.236	0.534 **	0.320 *	0.555 **	0.300	0.399 *	0.536 **
Tenderness	0.765 **	0.530 **	0.929 **	0.852 **	0.782 **	0.946 **	0.869 **	0.875 **
Juiciness	0.112	0.207	0.707 **	0.491 *	0.602 **	0.635 **	0.738 **	0.429 *
Perception of the degree of maturity	−0.805 **	0.056	−0.807 **	−0.840 **	−0.808 **	−0.819 **	−0.829 **	−0.885 **

* *p* < 0.05; ** *p* < 0.01.

**Table A4:** Pearson’s correlation coefficients between water phase salt (WPS) and sensory scores for the salami, calculated within storage times (fat inclusion, salt and starter cultures) and within treatments (10 and 20 weeks).

Sensory Attributes	Fat Inclusion (%)		Salt (%)		Starter Culture		Weeks	
	30	40	2.4	2.6	6	8	10	20
Color homogeneity	0.640 **	0.494 **	0.710 **	0.661 **	0.721 **	0.710 **	0.411 *	0.783 **
Cohesiveness	0.685 **	0.399 *	0.701 **	0.643 **	0.688 **	0.707 **	0.730 **	0.781 **
Odor intensity	−0.470 **	−0.425 *	−0.231	−0.653 **	−0.235	−0.694 **	0.291	−0.269
Odor rancidity	−0.359 *	0.089	−0.302	−0.476 **	−0.169	−0.397 *	0.214	−0473 **
Odor gamy	−0.762 **	−0.739 **	−0.656 **	−0.796 **	−0.679 **	−0.739 **	−0.519 **	−0.324
Odor spicy	−0.699 **	0.387 *	0.041	−0.295	−0.007	−0.088	0.462 **	−0.616 **
Flavor metallic	0.200	−0.129	−0.487 **	0.232	0.011	−0.232	−0.530 **	−0.316
Intensity off-flavor	−0.131	0.127	−0.417 *	−0.224	−0.447 *	−0.159	−0.435 *	−0.515 **
Tenderness	−0.817 **	−0.432 *	−0.769 **	−0.700 **	−0.613 **	−0.797 **	−0.728 **	−0.764 **
Juiciness	−0.119	−0.372 *	−0.504 **	−0.462 **	−0.403 *	−0.504 **	−0.698 **	−0.343
Perception of the degree of maturity	0.840 **	−0.037	0.664 **	0.698 **	0.686 **	0.713 **	0.792 **	0.769 **

* *p* < 0.05; ** *p* < 0.01.

**Table A5:** Factor loadings for each variable studied on the first two principal components.

Compounds	PC1	PC2	Communality
pH	0.714	−0.264	0.579
a_w_	−0.942	−0.056	0.891
Sodium chloride	0.817	0.266	0.738
Moisture:protein (M:P)	−0.934	−0.007	0.872
Water phase salt (WPS)	0.883	0.297	0.868
Alpha-tocopherol	0.629	−0.157	0.420
Free fatty acids (FFA)	0.563	0.682	0.782
Conjugated dienes (CD)	−0.624	−0.423	0.568
TBARs	0.253	−0.173	0.094
Non-protein nitrogen (NPN)	−0.719	0.238	0.574
External shape	−0.078	0.559	0.319
Casing adhesion	0.243	0.534	0.344
External mold	0.821	−0.101	0.684
Color homogeneity	0.738	−0.054	0.548
Cohesiveness	0.889	−0.291	0.875
Fat inclusion	−0.717	0.440	0.708
Odor intensity	−0.433	−0.302	0.279
Odor moldy	0.674	0.386	0.603
Odor rancidity	−0.492	0.192	0.279
Odor gamy	−0.728	−0.365	0.663
Odor spicy	−0.172	0.121	0.044
Flavor gamy	−0.584	−0.236	0.397
Flavor salty	0.425	0.449	0.382
Flavor rancidity	−0.177	0.620	0.416
Flavor metallic	−0.316	0.473	0.324
Flavor fatty	−0.447	0.795	0.832
Flavor moldy	0.052	0.700	0.493
Flavor spicy	−0.110	0.379	0.156
Flavor persistency	0.238	0.279	0.134
Intensity off-flavor	−0.479	0.493	0.472
Tenderness	−0.908	0.235	0.880
Juiciness	−0.600	0.174	0.390
Perception of the degree of maturity	0.873	−0.289	0.846
SFA	0.008	−0.751	0.564
MUFA	−0.784	0.156	0.639
PUFA	0.696	0.315	0.584
*n*-6	0.693	0.306	0.574
*n*-3	0.720	0.182	0.552
Eigenvalue	15.4	6.8	
Percentage of variance	39.5	17.5	
Cumulative percentage	39.5	57.0	
